# Mobile Health–Supported Virtual Reality and Group Problem Management Plus: Protocol for a Cluster Randomized Trial Among Urban Refugee and Displaced Youth in Kampala, Uganda (Tushirikiane4MH, Supporting Each Other for Mental Health)

**DOI:** 10.2196/42342

**Published:** 2022-12-08

**Authors:** Carmen H Logie, Moses Okumu, Jean-Luc Kortenaar, Lesley Gittings, Naimul Khan, Robert Hakiza, Daniel Kibuuka Musoke, Aidah Nakitende, Brenda Katisi, Peter Kyambadde, Torsum Khan, Richard Lester, Lawrence Mbuagbaw

**Affiliations:** 1 Factor-Inwentash Faculty of Social Work University of Toronto Toronto, ON Canada; 2 Women's College Research Institute Women's College Hospital Toronto, ON Canada; 3 Institute for Water, Environment & Health United Nations University Hamilton, ON Canada; 4 Centre for Gender & Sexual Health Equity Vancouver, BC Canada; 5 School of Social Work University of Illinois Urban-Champaign, IL United States; 6 School of Social Sciences Uganda Christian University Mukono Uganda; 7 Dalla Lana School of Public Health University of Toronto Toronto, ON Canada; 8 School of Health Studies Faculty of Health Sciences Western University London, ON Canada; 9 Centre for Social Science Research University of Cape Town Cape Town South Africa; 10 Electrical, Computer, and Biomedical Engineering Toronto Metropolitan University Toronto, ON Canada; 11 Young African Refugees for Integral Development Kampala Uganda; 12 International Research Consortium Kampala Uganda; 13 National AIDS and STI Control Programme Ministry of Health Kampala Uganda; 14 Most At Risk Population Initiative Mulago Hospital Kampala Uganda; 15 Department of Medicine University of British Columbia Vancouver, BC Canada; 16 Department of Health Research Methods, Evidence and Impact McMaster University Hamilton, ON Canada; 17 Department of Anesthesia McMaster University Hamilton, ON Canada; 18 Department of Pediatrics McMaster University Hamilton, ON Canada; 19 Biostatistics Unit Father Sean O'Sullivan Research Centre St Joseph's Healthcare Hamilton, ON Canada; 20 Centre for Development of Best Practices in Health Yaoundé Central Hospital Yaoundé Cameroon; 21 Division of Epidemiology and Biostatistics Department of Global Health Stellenbosch University Cape Town South Africa

**Keywords:** adolescents and youth, mental health, refugee, implementation research, virtual reality, mobile health, Uganda, urban

## Abstract

**Background:**

Although mental health challenges disproportionately affect people in humanitarian contexts, most refugee youth do not receive the mental health support needed. Uganda is the largest refugee-hosting nation in Africa, hosting over 1.58 million refugees in 2022, with more than 111,000 living in the city of Kampala. There is limited information about effective and feasible interventions to improve mental health outcomes and mental health literacy, and to reduce mental health stigma among urban refugee adolescents and youth in low- and middle-income countries (LMICs). Virtual reality (VR) is a promising approach to reduce stigma and improve mental health and coping, yet such interventions have not yet been tested in LMICs where most forcibly displaced people reside. Group Problem Management Plus (GPM+) is a scalable brief psychological transdiagnostic intervention for people experiencing a range of adversities, but has not been tested with adolescents and youth to date. Further, mobile health (mHealth) strategies have demonstrated promise in promoting mental health literacy.

**Objective:**

The aim of this study is to evaluate the feasibility and effectiveness of two youth-tailored mental health interventions (VR alone and VR combined with GMP+) in comparison with the standard of care in improving mental health outcomes among refugee and displaced youth aged 16-24 years in Kampala, Uganda.

**Methods:**

A three-arm cluster randomized controlled trial will be implemented across five informal settlements grouped into three sites, based on proximity, and randomized in a 1:1:1 design. Approximately 330 adolescents (110 per cluster) are enrolled and will be followed for approximately 16 weeks. Data will be collected at three time points: baseline enrollment, 8 weeks following enrollment, and 16 weeks after enrollment. Primary (depression) and secondary outcomes (mental health literacy, attitudes toward mental help–seeking, adaptive coping, mental health stigma, mental well-being, level of functioning) will be evaluated.

**Results:**

The study will be conducted in accordance with CONSORT (Consolidated Standards of Reporting Trials) guidelines. The study has received ethical approval from the University of Toronto (#40965; May 12, 2021), Mildmay Uganda Research Ethics Committee (MUREC-2021-41; June 24, 2021), and Uganda National Council for Science & Technology (SS1021ES; January 1, 2022). A qualitative formative phase was conducted using focus groups and in-depth, semistructured key informant interviews to understand contextual factors influencing mental well-being among urban refugee and displaced youth. Qualitative findings will inform the VR intervention, SMS text check-in messages, and the adaptation of GPM+. Intervention development was conducted in collaboration with refugee youth peer navigators. The trial launched in June 2022 and the final follow-up survey will be conducted in November 2022.

**Conclusions:**

This study will contribute to the knowledge of youth-tailored mental health intervention strategies for urban refugee and displaced youth living in informal settlements in LMIC contexts. Findings will be shared in peer-reviewed publications, conference presentations, and with community dissemination.

**Trial Registration:**

ClinicalTrials.gov NCT05187689; https://clinicaltrials.gov/ct2/show/NCT05187689

**International Registered Report Identifier (IRRID):**

DERR1-10.2196/42342

## Introduction

At the end of 2021, there were 89.4 million forcibly displaced persons across the globe, 83% of whom were hosted in low- and middle-income countries (LMICs) and 41% were children and youth under 18 years old [1]. Mental health challenges such as depression and anxiety disproportionately affect persons in humanitarian contexts; yet, most forcibly displaced persons do not receive the mental health support needed [2,3]. Myriad stressors contribute to psychological distress among refugee and displaced youth, including trauma, violence, food insecurity, and social marginalization [2,4]. Chronic psychological stress among adolescents can have life-long harmful impacts on neurobiological systems that are connected with emotional and behavioral regulation [5]. It is therefore of particular importance to mitigate psychological distress and to promote mental well-being among refugee and displaced adolescents and youth.

Uganda is the largest refugee-hosting nation in Africa with over 1.5 million refugees in 2022 [6]. There are 118,000 urban refugees in Uganda who live in Kampala, 27% of whom are youth aged 15-24 years [7]. There is a trend of urbanization of refugees globally, with more than 60% of refugees and 80% of internally displaced persons globally living in urban regions [8]. Many urban refugees such as those in Kampala live in informal settlements, including slums [9-11]. Socioenvironmental stressors in slums and informal settlements—such as violence and poverty—may harm mental well-being, yet mental health interventions in these contexts have not centered on the needs and priorities of urban refugee and displaced youth [12]. Most studies on the health of refugee and displaced persons have focused on refugee settlement contexts, leaving knowledge gaps regarding efficacious strategies to improve mental health among urban refugees [11].

A systematic review of mental health and psychosocial support for children (7-18 years old) in humanitarian settings identified knowledge gaps regarding effective strategies to reduce depression and anxiety [3]. Strategies in Uganda included sports for development, which only resulted in positive effects for boys [13], and a creative play group, which only resulted in positive effects for girls [14]. A 2018 cross-sectional survey with urban refugee and displaced youth (N=445) aged 16-24 years in Kampala, Uganda, found that three-quarters (74%) of adolescent girls and young women, and nearly half (49%) of adolescent boys and young men reported depressive symptoms [4]. These alarming statistics highlight the need for concrete solutions developed with and for urban refugee youth to alleviate this distress and risk of depression. In July 2020, Uganda opened its borders to receive more refugees from the Democratic Republic of the Congo (DRC) in the midst of the COVID-19 pandemic [15]. While mental health risks may be heightened during pandemics such as COVID-19 [16], researchers identified a chronically high prevalence of depression among urban refugee youth in Kampala both before (27.5%) and after (28.9%) declaration of the pandemic in March 2020 [17], signaling the urgent need to address chronic depression among this population.

Two key mental health needs that are understudied among refugee youth in LMICs such as Uganda include mental health literacy and mental health stigma reduction. Mental health literacy comprises knowledge and understanding of (1) different types of mental health issues and distress, (2) mental health risks and underlying causes of mental health challenges, (3) self-help strategies for mental health, (4) accessible and available professional help, and (5) recognizing when to seek mental health support and how to access it [18]. Interventions to advance mental health literacy may result in help-seeking for depression, anxiety, and psychological distress; however, there is a need for rigorous evaluations of the benefits of mental health literacy programs in LMIC and humanitarian contexts [19]. Researchers have called for more mental health literacy interventions specifically in Africa [20]. Indeed, mental health literacy programs tailored for refugees have largely focused on adults and/or high-income contexts [21,22]. This is also true for mental health stigma reduction, with the only randomized controlled trial (RCT) identified with refugees having been conducted with adult refugee men in the high-income context of Australia [23]. A systematic review reported that no trials have focused on reducing mental health stigma in low-income countries [24]. This review also reported a range of efficacious approaches to reducing mental health stigma, including via advancing mental health literacy [24]. Another systematic review on mental health stigma reduction reported that educational interventions were effective in reducing stigma, and that there were no differences in effectiveness between online and face-to-face mental health stigma reduction [25].

Emerging evidence suggests that tailored virtual reality (VR) scenarios can contribute to improved mental health outcomes. For example, a recent systematic review documented the efficacy of VR in treating posttraumatic stress disorder [26]. Yet, all studies were focused on adults in high-income settings and most were conducted with veterans [26]. To our knowledge, VR has not been piloted with youth in LMIC or humanitarian settings. Another promising mental health intervention approach is Group Problem Management Plus (GPM+), a World Health Organization (WHO) scalable group-based brief psychological transdiagnostic intervention for persons experiencing a range of adversities [27]. Problem Management Plus, delivered to individuals, was associated with reduced psychological distress, anxiety, depression, personally identified problems, and posttraumatic stress in RCTs with Kenyan adults [28] and conflict-affected adults in Pakistan [29]. The group-based delivery format, GPM+, was feasible and acceptable among conflict-affected adults in Nepal [30] and Pakistan [31], and is currently being tested among adult refugees in Turkey [32] and Jordan [33]. The WHO states that this program is likely efficacious among adolescents aged 16 years and older [27]; however, GPM+ has not yet been evaluated with refugee adolescents and youth.

This paper provides the study protocol for the Tushirikiane (roughly translating to “Supporting Each Other” in Swahili) for Mental Health (Tushirikiane4MH) study that aims to address these knowledge gaps regarding efficacious mental health interventions with and for urban refugee and displaced youth in Kampala, Uganda. Specifically, Tushirikiane4MH will test the effectiveness and feasibility of a VR intervention on its own and the combination of VR and GPM+ compared with standard of care. The findings can be used to inform the implementation and scale-up of mental health interventions with urban refugee and displaced youth across Uganda and other humanitarian contexts.

This study aims to evaluate the feasibility of two youth-tailored mental health interventions: (1) a VR experience focused on mental health literacy and psychological first-aid [34] skills, supplemented with mobile health (mHealth) delivered via SMS-based bidirectional messages and information; and (2) GPM+ in combination with the VR experience. A third arm, the waitlist control arm, will receive the GPM+ intervention on its own after the first two interventions are complete. The primary study objective is to determine the effectiveness of the interventions on reducing depression [35]. Secondary objectives include examining the effectiveness of the interventions on (1) improving mental health literacy [36-38], (2) increasing adaptive coping strategies [39,40], (3) reducing mental health stigma [41], (4) increasing mental health well-being [42], and (5) improving level of functioning [43].

## Methods

### Study Design

To evaluate intervention effectiveness, we will conduct a cluster randomized controlled trial (cRCT), in which five informal settlements in Kampala will be randomized at a 1:1:1 ratio to one of the three study arms. Peer navigators, refugee youth living in the five informal settlements, are trained in research methods and ethics, and will enroll youth into the study following obtaining written informed consent. Youth will be assigned to the study arm corresponding to the informal settlement they live in. A cluster randomization approach was selected to mitigate challenges of experimental contamination, as youth living in slums and informal settlements have shared sociophysical environments [44], thus addressing internal validity threats, although we will collect individual-level outcome data. We will perform data collection at three time points: baseline enrollment, 8 weeks following implementation, and 16 weeks following implementation.

### Study Setting

This trial is being conducted in the Ugandan capital of Kampala in five informal settlements, grouped into three sites based on geographic proximity (1: Kabalagala and Kansanga, 2: Katwe and Nsambya, and 3: Rubaga). These settlements were purposively chosen because: (1) they host many refugee and displaced persons in Kampala [45-48], largely from the DRC, Rwanda, and Burundi [49]; (2) these communities share similarities in socioeconomic status and living conditions, health care access, and languages; and (3) prior research noted a high prevalence of depressive symptoms among urban refugee youth in these communities [4,17]. Full details regarding the trial site geography and population have been described elsewhere [50].

### Study Population and Eligibility Criteria

Approximately 330 youth (110 per cluster) aged 16-25 years will be enrolled into this study. Eligibility criteria for inclusion are: (1) currently living in one of the five selected informal settlements in Kampala (Kabalagala, Kansanga, Katwe, Nsambya, or Rubaga); (2) identifying as a refugee or displaced person, or having refugee or displaced parents; (3) aged 16-25 years; (4) owning or have daily access to a mobile phone; and (5) speaking French, English, Luganda, Kirundi, Kinyarwanda, or Swahili. Eligibility screening (via phone, in person, or WhatsApp) with interested participants will be conducted by trained peer navigators.

### Participant Recruitment and Retention

The project team includes a refugee youth–focused community-based nongovernmental organization with expertise on youth engagement and programs for refugee youth. The team also includes academics, community-based practitioners, and stakeholders from the Ugandan Ministry of Health. Peer navigators—refugee and displaced youth aged 18-24 years living in these same five informal settlements (Kabalagala, Kansanga, Katwe, Nsambya, or Rubaga)—will work with the study coordinator and implementing partners to facilitate participant recruitment, and will further participate in study design and pilot testing. The 12 peer navigators (6 young women, 6 young men) have experience working in the various study communities as health educators or peer educators. They were identified and recruited by community-based collaborators and are deeply respected and connected in their communities.

Participants will be recruited within each settlement using purposive methods, including word-of-mouth and venue-based sampling at refugee agencies and community events. Recruitment will begin with participants who belong to the Tushirkiane cohort with this same study team and participated in previous completed trials on HIV self-testing [50] and COVID-19 prevention [51]. There will be additional purposive recruitment of 16- and 17-year-old participants to refresh the cohort. To engage and retain participants, peer navigators employed multiple strategies, including SMS and WhatsApp reminders, and local community partners also facilitated maintaining connection with study participants through outreach events.

### Patient and Public Involvement in Research

Study collaborators at Young African Refugees for Integral Development (YARID), a well-established youth refugee nongovernmental organization in Kampala, have been involved in the research from the initial stage of developing the research question and focus. The study protocol was developed after a formative qualitative research phase (Phase 1), which included: (1) in-depth semistructured key informant interviews with professionals in various roles supporting the health and well-being of refugee youth in Uganda (n=10); and (2) age- and- gender-segregated focus groups (4 discussions with 6 people in each focus group; n=24 participants in total) with refugee youth in Kampala aged 16-24 years. One focus group discussion each was held with young women aged 16-19 years, young women aged 20-24 years, young men aged 16-19 years, and young men aged 20-24 years, respectively. This formative qualitative work explored refugee youth perspectives on mental health, mental health literacy, and mental health stigma. These qualitative findings were used to identify key themes for development of the VR scenario, SMS content, and to adapt GPM+ to this context and population group. In this way, the study responds to the mental health needs and priorities of urban refugee youth in this context.

### Intervention Description

#### Design

**Figure 1:**
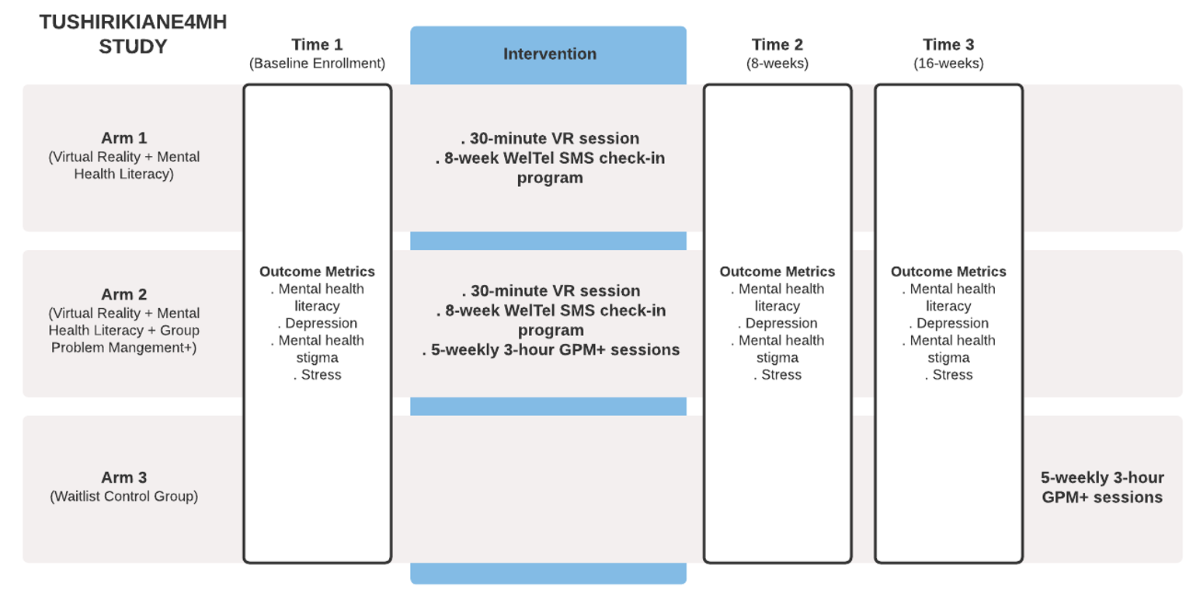
Study design for Tushirikiane4MH, a cluster randomized controlled trial of mental health literacy and mental health promotion strategies among urban refugee and displaced youth in Kampala, Uganda. GPM+, Group Problem Management Plus; VR: virtual reality.

The study was designed as a three-arm cRCT consisting of two treatment arms and one control arm. Clusters will be randomized to one of three arms: (1) VR, (2) VR plus GPM+, or (3) waitlist control, followed by GPM+ after intervention implementation and follow-up. Data will be collected at baseline, after intervention implementation (8 weeks postintervention), and at follow-up (16 weeks postintervention). The third arm (waitlist control) will receive an additional survey 8 weeks following the intervention to evaluate outcome changes. The VR and GPM+ arms receive weekly SMS messages with mental health literacy information and bidirectional check-ins (“how are you?” messages) where they can access any needed peer support. All study members will receive YARID resources and referrals as needed for mental health support from a trained social worker. The trial arms and interventions are described below and summarized in Figure 1.

#### Arm 1: VR Alone

Participants in this arm will be enrolled into an immersive, interactive 15-minute VR session. The VR scenario was designed to address Phase 1 findings, and integrates mental health literacy [18], psychological first aid [34], and mindful self-compassion information and activities [52]. The VR space was designed to be visibly similar to the environment of informal settlements in Kampala, and will be offered in participants’ language of choice (French, English, Kirundi, Kinyarwanda, or Swahili). Participants will be invited to participate in the VR scenario in a private room or in an outdoor setting at the partner organization. The VR session will be viewed on low-cost VR headsets and the study equipment will be sanitized and cleaned between uses.

#### Arm 2: VR and GPM+

Participants in this arm will be enrolled into the VR intervention (as described above) as well as GPM+. GPM+ is a WHO brief psychological transdiagnostic intervention for persons experiencing a range of adversities, including poverty and war, that, over five group sessions, aims to address both practical (eg, housing) and emotional (eg, stress) challenges [27]. Sessions employ evidence-based approaches to stress management, problem-solving, behavioral activation, and social support to reduce a range of mental health concerns. The intervention includes five 3-hour sessions, each with a distinct mechanism of action, which are described in Table 1.

**Table 1:** Group Problem Management Plus sessions and their mechanisms of action.

Session	Key mechanisms of action
Managing stress	Identifying goals, learning deep breathing and techniques for stress management
Managing problems	Identifying one solvable and practical problem, brainstorming possible solutions together
Get going, keep doing	Learning about depression and inactivity, and identifying and planning small enjoyable activities
Strengthening social support	Discussing a range of social support resources and making plans to increase social support
Staying well	Reviewing all of the mechanisms of action in the prior four sessions

Training materials and content were adapted with peer navigators and based on formative qualitative findings to enhance relevance to urban refugee youth in Kampala. The WHO suggests making small changes to case examples such as relatable problems and social support–seeking examples. Peer navigators will deliver the five group sessions with groups of up to 20 participants.

We will provide participants in Arm 1 and Arm 2 with additional mHealth support, including SMS bidirectional check-ins, SMS mental health literacy messages, and supportive WhatsApp conversations. The aim of this mHealth support is to ensure that any participant in the VR/GMP+ group who needs additional mental health support or information will receive it, and that mHealth will also reinforce the information and access to resources shared in the intervention arms. The SMS program includes weekly SMS blasts designed to reinforce information provided in the VR on mental health literacy [18], psychological first-aid information and skills [34], and self-compassion activities [52]. There are also weekly bidirectional check-ins asking participants “how are you?” in their preferred language; any participant who responds they are not well or requests help will be referred within 48 hours to a social worker based at YARID as well as their peer navigator. This SMS platform is hosted by WelTel [53,54], a nonprofit agency [55]. The WelTel system will manage this bidirectional SMS intervention on their structured mobile phone platform on which all SMS interactions will be logged. To provide additional group-based support to participants, we will invite Arm 1 and Arm 2 participants to take part in weekly WhatsApp group discussions between peer navigators with ~25 participants assigned to each peer navigator. These discussions will be moderated by peer navigators, a research assistant, and a trained research coordinator, and will address the same content regarding mental health literacy, stigma, and stress-coping strategies covered in the VR intervention.

#### Arm 3: Waitlist Control

Participants in this arm will be waitlisted and receive the GPM+ intervention after implementation and follow-up. The waitlist control group will complete a fourth survey at 8 weeks to evaluate changes after participating in GPM+. In the meantime, the waitlist control group will have access to YARID resources and referrals as needed for mental health support.

### Outcomes

#### Primary Outcome

The primary outcome measured in this trial is *depression*, which will be measured using the Patient Health Questionnaire-9 [56].

#### Secondary Outcomes

Secondary outcomes include (1) *mental health literacy*, assessed with a modified depression literacy scale validated in LMICs [36-38]; (2) *attitudes toward mental health help–seeking*, assessed with the Inventory of Attitudes Towards Seeking Mental Health Services [57]; (3) *adaptive coping strategies*, assessed with the Kidcope [39] and Self-Compassion Scale for Youth [40]; (4) *mental health stigma*, measured using the Brief Version of the Internalized Stigma of Mental Illness scale [41]; (5) *mental well-being*, assessed with the WHO-Five Wellbeing Scale [42]; and (6) *level of functioning*, measured using the WHO Disability Assessment Schedule [43].

### Sample Size and Power Analysis

The study aims to include 330 participants, with 110 per study arm. Cluster sizes of 90 per group (n=270) are required to have 80% power (*P*<.05) to detect a difference of ~3 points in mean depression score (moderate effect size) at a level of significance of α=.05, assuming an intraclass correlation of 0.01 and SD of 7. With 10% attrition, 297 participants (99 per cluster) are required. Computations were performed using RStudio version 3.3.0, based on formulae for multiple comparisons of proportions and adjusted for design effect [58].

### Data Collection and Management

Data will be collected using a structured survey on cell phones or tablets in all study languages by trained research assistants using the SurveyCTO app (Dobility), a secure platform that automatically encrypts data, which will be uploaded using a Secure Sockets Layer (SSL) certificate to a password-protected server. SurveyCTO allows for offline data collection, facilitates multilingual data collection, and has branching logic and consistency checks. To enhance confidentiality, all participants will be assigned a unique participant ID and no personal identifying information will be collected. Only study staff will have access to the data set on a need-to-know basis for the purpose of data management and outcome reporting. All data sets will be saved on a password-protected server.

### Data Analysis Plan

Analysis and reporting for the cRCT (Phase 2) will be conducted in accordance with CONSORT (Consolidated Standards of Reporting Trials) guidelines [59] (Multimedia Appendix 1). The analyst will be blinded to group allocation. A flow diagram will be used to illustrate patient flow (screening, randomization, allocation, follow-up). Baseline data will be reported for all three groups and summarized as mean (SD) or median (IQR) for continuous variables, and as counts and number (%) for categorical variables. The primary analysis will be intention-to-treat analysis (data from participants will be analyzed according to their allocation, irrespective of whether they actually received that intervention).

We will perform multivariable regression analyses, adjusting for the outcome measure at baseline and stratification variables. For the primary analysis to assess differences among the three intervention conditions on the outcomes, indicator variables will include intervention assignment and a vector of baseline covariates (eg, sociodemographics). Analyses will adjust for multiple comparisons across the three intervention conditions using the Fisher protected least-significant difference test, first assessing differences between intervention groups and, if the Omnibus *F* test is significant, subsequently calculating pairwise comparisons.

Between-group comparisons will be performed using multilevel mixed-effects logistic or linear regression models (to account for clustering) depending on which outcome is being evaluated. For these models, the intervention group will be entered as a fixed effect. The significance level will be set at α=.05. The results will be expressed as odds ratios or mean differences, as appropriate, accompanied by 95% CIs and *P* values. We will perform an adjusted analysis for the primary outcomes to investigate the role of various covariates on the relative effect. Covariates (eg, age) will be entered as a block. We will explore gender differences in primary and secondary intervention outcomes. Given that the outcomes of this study are related to behavior change and the trial is of a short duration with minimal risks, a data monitoring committee was not deemed necessary.

### Ethical Considerations

The Tushirikiane4MH trial protocol has been approved by the Research Ethics Boards University of Toronto (May 12, 2021), Mildmay Uganda Research Ethics Committee (June 24, 2021), and Uganda National Council for Science & Technology (January 6, 2022). The trial is registered at ClinicalTrials.gov (NCT05187689).

The protocol for the study was developed in accordance with the SPIRIT (Standard Protocol Items: Recommendations for Interventional Trials) Statement [60,61]. The study population includes young adults (aged 16 years and over) capable of providing informed consent. We received ethics approval to allow youth aged 16-17 years to participate without parental consent; this is a common approach to reduce barriers to youth participation in health research on potentially sensitive topics [62,63].

All participants will receive information about the study before being enrolled to ensure understanding of rights for refusal/withdrawal, study processes, and expectations. To ensure the protection of human subjects, all participants will be provided with sufficient time to give written voluntary consent to participate in the study. All informed written consent processes will occur in a private room at a location provided by YARID. The participant will read the consent form themselves or a peer navigator will read the informed consent text aloud in a language comfortable to the participant (French, English, Luganda, Kirundi, Kinyarwanda, or Swahili) and will ask if the participant has any questions and will answer their questions. A peer navigator will ask the participants to sign the consent form or provide a thumbprint to indicate their consent. The consent form will in no way be connected with data collected and will be destroyed 5 years after data collection is completed.

At any time during the study data collection period, participants can withdraw from the study before completing the interview with no adverse consequences on the care or services they receive. All data will be stored on password-protected computers. To maintain confidentiality, all participants will be given a unique Case ID, and no personal identifying information will be stored with the study data.

The risks associated with the Tushirikiane4MH trial are reasonable. All intervention components have been designed as youth-friendly, based on the principles of psychological first aid and evidence with adult populations. Qualitative findings informed intervention design and the interventions were piloted with youth peer navigators. Although these interventions are not expected to cause psychological distress, this risk will be shared with participants. Peer navigators have been trained in psychological first aid, and trained counselors will be on site throughout the intervention. All participants will also be provided with a list of community resources.

Any adverse event will be reported by the peer navigators to the research assistant, who will fill out an Adverse Event Reporting Form and Adverse Event Narrative Form if appropriate. Adverse events can also be directly reported by study participants to YARID and the study team. Any adverse event requiring a narrative form will be reported to the principal investigators within 24 hours.

### Data Sharing

The final data set will be shared between the Uganda-based research team and members of the Toronto-based research team via a secured, encrypted, and password-protected system. The final deidentified data set will be available to users who enter a data-sharing agreement and secure research ethics approval via a research ethics board amendment with the University of Toronto.

## Results

The VR scenario, WelTel SMS content, and adaptation of GPM+ were conducted between January and May 2022. Research staff, including peer navigators, were trained in VR use, mental health literacy, and GPM+ in March 2022 and May 2022 in person, and virtually from May to June 2022. Baseline data collection was conducted in April 2022 and the intervention was conducted in June 2022. The first follow-up survey was conducted in August 2022 and the final follow-up survey will be conducted in November 2022. Any important protocol modifications will be included as amendments in Research Ethics Board submissions and updated on ClinicalTrials.gov.

## Discussion

### Projected Significance

Trial findings will generate novel evidence on promising low-cost mental health interventions delivered by and for refugee and displaced youth. Specifically, we anticipate finding that VR and GPM+ conducted on their own are feasible and will each be associated with improved mental health outcomes in comparison with standard of care. We also anticipate finding that when conducted in tandem, VR and GPM+ will be associated with greater improvements in mental health compared to when they are conducted separately and when compared with the standard of care. Importantly, the findings will enhance understanding of how VR interventions, effective in supporting adult mental health in high-income contexts [26], could be effective among urban refugee youth in LMICs. Study findings will also advance knowledge of how the WHO GPM+, effective with adults [30,31], could potentially benefit adolescents aged 16 years and older [27]. In sum, the findings will build on the limited evidence base of interventions to improve refugee youth mental well-being [3].

By measuring the effectiveness and feasibility of these novel, low-cost mental health innovations, this study has the potential to inform research, policy, and practice in the health, education, and social development sectors. Community partners and knowledge users will be involved in all stages of trial design, conduct, analysis, and dissemination. Findings regarding the effectiveness of GPM+ or VR in supporting mental health outcomes of refugee and displaced youth can inform the scale-up of such mental health interventions for other refugee youth in Uganda—both urban- and refugee settlement–based—as well as other urban forcibly displaced youth in East Africa.

### Dissemination

Irrespective of the study findings, trial results will be published in peer-reviewed scientific journals following international authorship guidelines, and will be presented to academics and researchers at key scientific conferences. The findings will be disseminated through a variety of methods, including community reports and policy briefs, to a range of stakeholders, including academics and researchers in mental health, international collaborating organizations such as the United Nations High Commissioner for Refugees, Ugandan Ministries of Health and Education, implementing partners in adolescent health and social work, and refugee community organizations and members. We will also create a short video on how to implement the findings into practice, as well as a graphic novel depicting the intervention and findings.

### Strengths and Limitations

Strengths of this study include its focus on identifying effective intervention approaches for improving mental health among urban refugee and displaced youth in Kampala, Uganda, an understudied population in mental health research. Another strength is adapting and evaluating evidence-based approaches for a different context (eg, adapting VR for the LMIC context) and different age groups (eg, adapting GPM+ for refugee youth). Finally, our study will conduct gender- and age-stratified analyses, providing insight into gender or age differences in intervention effectiveness. The primary study limitations are loss to follow-up due to the mobile nature of the population and resulting missing data points.

## Appendix

Multimedia Appendix 1 CONSORT-EHEALTH checklist (V.1.6.1).

## Abbreviations

CONSORT: Consolidated Standards of Reporting Trials
cRCT: cluster randomized controlled trial
DRC: Democratic Republic of Congo
GPM+: group problem management plus
LMIC: low- and- middle-income country
mHealth: mobile health
RCT: randomized controlled trial
SPIRIT: Standard Protocol Items: Recommendations for Interventional Trials
SSL: Secure Sockets Layer
VR: virtual reality
WHO: World Health Organization
YARID: Young African Refugees for Integral Development
