# Supplementary material for: Mobile Health–Supported Virtual Reality and Group Problem Management Plus: Protocol for a Cluster Randomized Trial Among Urban Refugee and Displaced Youth in Kampala, Uganda (Tushirikiane4MH, Supporting Each Other for Mental Health)
Source: JMIR Res Protoc. 2022 Dec 8;11(12):e42342. doi: 10.2196/42342 (PMC9782374; doi:10.2196/42342)
Supplement: Multimedia Appendix 1 [file resprot_v11i12e42342_app1.pdf]

CONSORT-EHEALTH (V 1.6.1) - Submission/Publication Form

The CONSORT-EHEALTH checklist is intended for authors of randomized trials evaluating web-based and Internet-based applications/interventions, including mobile interventions, electronic games (incl multiplayer games), social media, certain telehealth applications, and other interactive and/or networked electronic applications. Some of the items (e.g. all subitems under item 5 - description of the intervention) may also be applicable for other study designs.

The goal of the CONSORT EHEALTH checklist and guideline is to be
a) a guide for reporting for authors of RCTs,
b) to form a basis for appraisal of an ehealth trial (in terms of validity)

CONSORT-EHEALTH items/subitems are MANDATORY reporting items for studies published in the Journal of Medical Internet Research and other journals / scientific societies endorsing the checklist.

Items numbered 1., 2., 3., 4a., 4b etc are original CONSORT or CONSORT-NPT (non-pharmacologic treatment) items.
Items with Roman numerals (i., ii, iii, iv etc.) are CONSORT-EHEALTH extensions/clarifications.

As the CONSORT-EHEALTH checklist is still considered in a formative stage, we would ask that you also RATE ON A SCALE OF 1-5 how important/useful you feel each item is FOR THE PURPOSE OF THE CHECKLIST and reporting guideline (optional).

Mandatory reporting items are marked with a red *.
In the textboxes, either copy & paste the relevant sections from your manuscript into this form - please include any quotes from your manuscript in QUOTATION MARKS,
or answer directly by providing additional information not in the manuscript, or elaborating on why the item was not relevant for this study.

YOUR ANSWERS WILL BE PUBLISHED AS A SUPPLEMENTARY FILE TO YOUR PUBLICATION IN JMIR AND ARE CONSIDERED PART OF YOUR PUBLICATION (IF ACCEPTED).
Please fill in these questions diligently. Information will not be copyedited, so please use proper spelling and grammar, use correct capitalization, and avoid abbreviations.

DO NOT FORGET TO SAVE AS PDF _AND_ CLICK THE SUBMIT BUTTON SO YOUR ANSWERS ARE IN OUR DATABASE !!!

Citation Suggestion (if you append the pdf as Appendix we suggest to cite this paper in the caption):
Eysenbach G, CONSORT-EHEALTH Group
CONSORT-EHEALTH: Improving and Standardizing Evaluation Reports of Web-based and Mobile Health Interventions
J Med Internet Res 2011;13(4):e126
URL: http://www.jmir.org/2011/4/e126/
doi: 10.2196/jmir.1923
PMID: 22209829

* Required

Your name *
First Last

Carmen Logie

Primary Affiliation (short), City, Country *

University of Toronto, Toronto, Canada

University of Toronto

Your e-mail address *

abc@gmail.com

carmen.logie@utoronto.ca

Title of your manuscript *

Provide the (draft) title of your manuscript.

Mental Health Literacy and Mental Health Promotion Among Urban Refugee and Displaced Youth in Kampala, Uganda: Protocol for a Mobile Health-Supported Virtual Reality and Group Problem Management Plus Cluster Randomized Trial (Tushirikiane 4MH, Supporting Each Othe for Mental Health)

Article Preparation Status/Stage *

At which stage in your article preparation are you currently (at the time you fill in this form)

- (●) not submitted yet - in early draft status
- ( ) not submitted yet - in late draft status, just before submission
- ( ) submitted to a journal but not reviewed yet
- ( ) submitted to a journal and after receiving initial reviewer comments
- ( ) submitted to a journal and accepted, but not published yet
- ( ) published
- ( ) Other:

Journal *

If you already know where you will submit this paper (or if it is already submitted), please provide the journal name (if it is not JMIR, provide the journal name under "other")

- ( ) not submitted yet / unclear where I will submit this
- (●) Journal of Medical Internet Research (JMIR)
- ( ) Other:

Manuscript tracking number *

If this is a JMIR submission, please provide the manuscript tracking number under "other" (The ms tracking number can be found in the submission acknowledgement email, or when you login as author in JMIR. If the paper is already published in JMIR, then the ms tracking number is the four-digit number at the end of the DOI, to be found at the bottom of each published article in JMIR)

- (●) no ms number (yet) / not (yet) submitted to / published in JMIR
- ( ) Other:

TITLE AND ABSTRACT

1a) TITLE: Identification as a randomized trial in the title

1a) Does your paper address CONSORT item 1a? *

I.e does the title contain the phrase "Randomized Controlled Trial"? (if not, explain the reason under "other")

(•) yes

( ) Other:

1a-i) Identify the mode of delivery in the title

Identify the mode of delivery. Preferably use "web-based" and/or "mobile" and/or "electronic game" in the title. Avoid ambiguous terms like "online", "virtual", "interactive". Use "Internet-based" only if Intervention includes non-web-based Internet components (e.g. email), use "computer-based" or "electronic" only if offline products are used. Use "virtual" only in the context of "virtual reality" (3-D worlds). Use "online" only in the context of "online support groups". Complement or substitute product names with broader terms for the class of products (such as "mobile" or "smart phone" instead of "iphone"), especially if the application runs on different platforms.

	1	2	3	4	5	
subitem not at all important	( )	( )	( )	(•)	( )	essential

Does your paper address subitem 1a-i? *

Copy and paste relevant sections from manuscript title (include quotes in quotation marks "like this" to indicate direct quotes from your manuscript), or elaborate on this item by providing additional information not in the ms, or briefly explain why the item is not applicable/relevant for your study

"Mobile Health-Supported"

1a-ii) Non-web-based components or important co-interventions in title

Mention non-web-based components or important co-interventions in title, if any (e.g., "with telephone support").

	1	2	3	4	5	
subitem not at all important	( )	( )	( )	(•)	( )	essential

Does your paper address subitem 1a-ii?

Copy and paste relevant sections from manuscript title (include quotes in quotation marks "like this" to indicate direct quotes from your manuscript), or elaborate on this item by providing additional information not in the ms, or briefly explain why the item is not applicable/relevant for your study

"Group problem management plus" and "Virtual Reality"
Group Problem Management Plus (GPM+) is a WHO scalable brief psychological transdiagnostic intervention for persons experiencing a range of adversities.

1a-iii) Primary condition or target group in the title

Mention primary condition or target group in the title, if any (e.g., "for children with Type I Diabetes") Example: A Web-based and Mobile Intervention with Telephone Support for Children with Type I Diabetes: Randomized Controlled Trial

	1	2	3	4	5	
subitem not at all important	○	○	○	○	⊙	essential

Does your paper address subitem 1a-iii? *

Copy and paste relevant sections from manuscript title (include quotes in quotation marks "like this" to indicate direct quotes from your manuscript), or elaborate on this item by providing additional information not in the ms, or briefly explain why the item is not applicable/relevant for your study

"Mental Health Literacy and Mental Health Promotion Among Urban Refugee and Displaced Youth in Kampala, Uganda"

1b) ABSTRACT: Structured summary of trial design, methods, results, and conclusions

NPT extension: Description of experimental treatment, comparator, care providers, centers, and blinding status.

1b-i) Key features/functionalities/components of the intervention and comparator in the METHODS section of the ABSTRACT

Mention key features/functionalities/components of the intervention and comparator in the abstract. If possible, also mention theories and principles used for designing the site. Keep in mind the needs of systematic reviewers and indexers by including important synonyms. (Note: Only report in the abstract what the main paper is reporting. If this information is missing from the main body of text, consider adding it)

	1	2	3	4	5	
subitem not at all important	○	○	○	⊙	○	essential

Does your paper address subitem 1b-i? *

Copy and paste relevant sections from the manuscript abstract (include quotes in quotation marks "like this" to indicate direct quotes from your manuscript), or elaborate on this item by providing additional information not in the ms, or briefly explain why the item is not applicable/relevant for your study

yes but in objective: "This study aims to evaluate the feasibility and effectiveness of youth-tailored, mHealth-supported mental health interventions among refugee and displaced youth aged 16-24 years in Kampala, Uganda. It will evaluate two strategies: a virtual reality experience, and an adapted version of the WHO's GPM+. Both interventions will supported by mobile health (mHealth) SMS check-ins.

1b-ii) Level of human involvement in the METHODS section of the ABSTRACT

Clarify the level of human involvement in the abstract, e.g., use phrases like “fully automated” vs. “therapist/nurse/care provider/physician-assisted” (mention number and expertise of providers involved, if any). (Note: Only report in the abstract what the main paper is reporting. If this information is missing from the main body of text, consider adding it)

	1	2	3	4	5	
subitem not at all important	○	○	○	◉	○	essential

Does your paper address subitem 1b-ii?

Copy and paste relevant sections from the manuscript abstract (include quotes in quotation marks "like this" to indicate direct quotes from your manuscript), or elaborate on this item by providing additional information not in the ms, or briefly explain why the item is not applicable/relevant for your study

This study will be implemented by peer navigators, with the support of a study coordinator.

1b-iii) Open vs. closed, web-based (self-assessment) vs. face-to-face assessments in the METHODS section of the ABSTRACT

Mention how participants were recruited (online vs. offline), e.g., from an open access website or from a clinic or a closed online user group (closed usergroup trial), and clarify if this was a purely web-based trial, or there were face-to-face components (as part of the intervention or for assessment). Clearly say if outcomes were self-assessed through questionnaires (as common in web-based trials). Note: In traditional offline trials, an open trial (open-label trial) is a type of clinical trial in which both the researchers and participants know which treatment is being administered. To avoid confusion, use “blinded” or “unblinded” to indicated the level of blinding instead of “open”, as “open” in web-based trials usually refers to “open access” (i.e. participants can self-enrol). (Note: Only report in the abstract what the main paper is reporting. If this information is missing from the main body of text, consider adding it)

	1	2	3	4	5	
subitem not at all important	◉	○	○	○	○	essential

Does your paper address subitem 1b-iii?

Copy and paste relevant sections from the manuscript abstract (include quotes in quotation marks "like this" to indicate direct quotes from your manuscript), or elaborate on this item by providing additional information not in the ms, or briefly explain why the item is not applicable/relevant for your study

A three-arm cluster randomized controlled trial will be implemented across five informal settlements grouped into three sites, based on proximity, and randomization will be performed with a 1:1:1 method.

1b-iv) RESULTS section in abstract must contain use data

Report number of participants enrolled/assessed in each group, the use/uptake of the intervention (e.g., attrition/adherence metrics, use over time, number of logins etc.), in addition to primary/secondary outcomes. (Note: Only report in the abstract what the main paper is reporting. If this information is missing from the main body of text, consider adding it)

	1	2	3	4	5	
subitem not at all important	(•)	( )	( )	( )	( )	essential

Does your paper address subitem 1b-iv?

Copy and paste relevant sections from the manuscript abstract (include quotes in quotation marks "like this" to indicate direct quotes from your manuscript), or elaborate on this item by providing additional information not in the ms, or briefly explain why the item is not applicable/relevant for your study

No. This is a protocol paper.

1b-v) CONCLUSIONS/DISCUSSION in abstract for negative trials

Conclusions/Discussions in abstract for negative trials: Discuss the primary outcome - if the trial is negative (primary outcome not changed), and the intervention was not used, discuss whether negative results are attributable to lack of uptake and discuss reasons. (Note: Only report in the abstract what the main paper is reporting. If this information is missing from the main body of text, consider adding it)

	1	2	3	4	5	
subitem not at all important	(•)	( )	( )	( )	( )	essential

Does your paper address subitem 1b-v?

Copy and paste relevant sections from the manuscript abstract (include quotes in quotation marks "like this" to indicate direct quotes from your manuscript), or elaborate on this item by providing additional information not in the ms, or briefly explain why the item is not applicable/relevant for your study

No. This is a protocol paper.

INTRODUCTION

2a) In INTRODUCTION: Scientific background and explanation of rationale

2a-i) Problem and the type of system/solution

Describe the problem and the type of system/solution that is object of the study: intended as stand-alone intervention vs. incorporated in broader health care program? Intended for a particular patient population? Goals of the intervention, e.g., being more cost-effective to other interventions, replace or complement other solutions? (Note: Details about the intervention are provided in "Methods" under 5)

	1	2	3	4	5	
subitem not at all important	○	○	●	○	○	essential

Does your paper address subitem 2a-i? *

Copy and paste relevant sections from the manuscript (include quotes in quotation marks "like this" to indicate direct quotes from your manuscript), or elaborate on this item by providing additional information not in the ms, or briefly explain why the item is not applicable/relevant for your study

Virtual Reality (VR) scenarios can lead to improved mental health. For example, a recent systematic review documented the efficacy of VR in treating PTSD [23]. Yet, all studies were focused on adults in high-income settings and most were focused on soldiers [23]. To our knowledge, VR has not been piloted with youth in low-resource and humanitarian settings.
Group Problem Management Plus (GPM+) is a WHO scalable brief psychological transdiagnostic intervention for persons experiencing a range of adversities [24]. In randomized controlled trials with Kenyan adults [25], and conflict affected adults in Pakistan [26] PM+ was associated with reduced psychological distress, anxiety, depression, personally identified problems, and post-traumatic stress. Group-PM+ was found to be feasible and acceptable for conflict affected adults in Nepal [27] and Pakistan [28], and is being tested with adult refugees in Turkey [29] and Jordan [30]. The WHO states that this program is likely efficacious among adolescents 16 and older [24]. However, Group-PM+ has not yet been implemented with refugee adolescents or youth.

2a-ii) Scientific background, rationale: What is known about the (type of) system

Scientific background, rationale: What is known about the (type of) system that is the object of the study (be sure to discuss the use of similar systems for other conditions/diagnoses, if appropiate), motivation for the study, i.e. what are the reasons for and what is the context for this specific study, from which stakeholder viewpoint is the study performed, potential impact of findings [2]. Briefly justify the choice of the comparator.

	1	2	3	4	5	
subitem not at all important	○	○	●	○	○	essential

Does your paper address subitem 2a-ii? *

Copy and paste relevant sections from the manuscript (include quotes in quotation marks "like this" to indicate direct quotes from your manuscript), or elaborate on this item by providing additional information not in the ms, or briefly

explain why the item is not applicable/relevant for your study

Virtual Reality (VR) scenarios can lead to improved mental health. For example, a recent systematic review documented the efficacy of VR in treating PTSD [23]. Yet, all studies were focused on adults in high-income settings and most were focused on soldiers [23]. To our knowledge, VR has not been piloted with youth in low-resource and humanitarian settings.
Group Problem Management Plus (GPM+) is a WHO scalable brief psychological transdiagnostic intervention for persons experiencing a range of adversities [24]. In randomized controlled trials with Kenyan adults [25], and conflict affected adults in Pakistan [26] PM+ was associated with reduced psychological distress, anxiety, depression, personally identified problems, and post-traumatic stress. Group-PM+ was found to be feasible and acceptable for conflict affected adults in Nepal [27] and Pakistan [28], and is being tested with adult refugees in Turkey [29] and Jordan [30]. The WHO states that this program is likely efficacious among adolescents 16 and older [24]. However, Group-PM+ has not yet been implemented with refugee adolescents or youth.

2b) In INTRODUCTION: Specific objectives or hypotheses

Does your paper address CONSORT subitem 2b? *

Copy and paste relevant sections from the manuscript (include quotes in quotation marks "like this" to indicate direct quotes from your manuscript), or elaborate on this item by providing additional information not in the ms, or briefly explain why the item is not applicable/relevant for your study

The specific objectives of this study are to determine the effectiveness of the interventions on the following criteria: (1) improved mental health literacy [31–33]; (2) reduced depression [34]; (3) adaptive coping strategies [35,36]; (4) reduced mental health stigma [37]; (5) mental health wellbeing [38]; and, (6) level of functioning [39]. Secondary outcomes are related to the implementation and feasibility of the intervention, including: (1) usage of usage of intervention by youth to improve mental health; (2) facilities/sites implementing the intervention; (3) intermediaries trained on how to use interventions; and, (4) usage of intervention by intermediaries to improve mental health in the community.

METHODS

3a) Description of trial design (such as parallel, factorial) including allocation ratio

Does your paper address CONSORT subitem 3a? *

Copy and paste relevant sections from the manuscript (include quotes in quotation marks "like this" to indicate direct quotes from your manuscript), or elaborate on this item by providing additional information not in the ms, or briefly explain why the item is not applicable/relevant for your study

"To evaluate intervention effectiveness, we will conduct a cluster randomized controlled trial (cRCT). The clusters include five informal settlements grouped into three sites that are randomized in a 1:1:1 method to one of three study arms. Once the youth have provided informed written consent they will be enrolled into the study and assigned to a study arm based on the community they live in. Although outcome data will be collected at the level of the individual, we selected cluster randomization over individual randomization because the intervention is implemented at the settlement level. A cluster randomized design addresses threats of internal validity. It reduces the possibility of experimental contamination due to the shared social and physical environments between youth in the same or nearby informal settlements [40]."

3b) Important changes to methods after trial commencement (such as eligibility criteria), with reasons

Does your paper address CONSORT subitem 3b? *

Copy and paste relevant sections from the manuscript (include quotes in quotation marks "like this" to indicate direct

quotes from your manuscript), or elaborate on this item by providing additional information not in the ms, or briefly explain why the item is not applicable/relevant for your study

No: it is a protocol

3b-i) Bug fixes, Downtimes, Content Changes

Bug fixes, Downtimes, Content Changes: ehealth systems are often dynamic systems. A description of changes to methods therefore also includes important changes made on the intervention or comparator during the trial (e.g., major bug fixes or changes in the functionality or content) (5-iii) and other "unexpected events" that may have influenced study design such as staff changes, system failures/downtimes, etc. [2].

	1	2	3	4	5	
subitem not at all important	(•)	( )	( )	( )	( )	essential

Does your paper address subitem 3b-i?

Copy and paste relevant sections from the manuscript (include quotes in quotation marks "like this" to indicate direct quotes from your manuscript), or elaborate on this item by providing additional information not in the ms, or briefly explain why the item is not applicable/relevant for your study

No, it is not relevant as this is a protocol paper

4a) Eligibility criteria for participants

Does your paper address CONSORT subitem 4a? *

Copy and paste relevant sections from the manuscript (include quotes in quotation marks "like this" to indicate direct quotes from your manuscript), or elaborate on this item by providing additional information not in the ms, or briefly explain why the item is not applicable/relevant for your study

"Individuals are eligible for inclusion if they meet the following criteria: (1) currently live in one of the following five informal settlements in Kampala: Kabalagala, Kansanga, Katwe, Nsambya, or Rubaga; (2) self-identify as a woman or man (transgender inclusive); (3) identify as a refugee/displaced person or have refugee/displaced parents; (4) are aged 16-25 years; (5) own a mobile phone; and, (6) speak French, English, Luganda, Kirundi, Kinyarwanda or Swahili."

4a-i) Computer / Internet literacy

Computer / Internet literacy is often an implicit "de facto" eligibility criterion - this should be explicitly clarified.

	1	2	3	4	5	
subitem not at all important	(•)	( )	( )	( )	( )	essential

Does your paper address subitem 4a-i?

Copy and paste relevant sections from the manuscript (include quotes in quotation marks "like this" to indicate direct quotes from your manuscript), or elaborate on this item by providing additional information not in the ms, or briefly explain why the item is not applicable/relevant for your study

No, it is not needed. but what is needed is mobile phone ownership that we include as stated above:
"Individuals are eligible for inclusion if they meet the following criteria: (1) currently live in one of the following five informal settlements in Kampala: Kabalagala, Kansanga, Katwe, Nsambya, or Rubaga; (2) self-identify as a woman or man (transgender inclusive); (3) identify as a refugee/displaced person or have refugee/displaced parents; (4) are aged 16-25 years; (5) own a mobile phone; and, (6) speak French, English, Luganda, Kirundi, Kinyarwanda or Swahili."

4a-ii) Open vs. closed, web-based vs. face-to-face assessments:

Open vs. closed, web-based vs. face-to-face assessments: Mention how participants were recruited (online vs. offline), e.g., from an open access website or from a clinic, and clarify if this was a purely web-based trial, or there were face-to-face components (as part of the intervention or for assessment), i.e., to what degree got the study team to know the participant. In online-only trials, clarify if participants were quasi-anonymous and whether having multiple identities was possible or whether technical or logistical measures (e.g., cookies, email confirmation, phone calls) were used to detect/prevent these.

	1	2	3	4	5	
subitem not at all important	( )	(•)	( )	( )	( )	essential

Does your paper address subitem 4a-ii? *

Copy and paste relevant sections from the manuscript (include quotes in quotation marks "like this" to indicate direct quotes from your manuscript), or elaborate on this item by providing additional information not in the ms, or briefly explain why the item is not applicable/relevant for your study

"Participants will be recruited within each settlement using purposive methods, including word-of-mouth and venue-based sampling at refugee agencies and community events. Priority will given to participants that participated in previous trials with the same research group on HIV self-testing and COVID-19 [45]. There will be additional purposive recruitment of 16 and 17 year old participants in order to renew the cohort. Peer navigators will facilitate participant retention and maintain engagement in the study using multiple study reminder strategies including SMS reminders and visits. We will utilize existing outreach and services by local refugee agencies and community partners."

4a-iii) Information giving during recruitment

Information given during recruitment. Specify how participants were briefed for recruitment and in the informed consent procedures (e.g., publish the informed consent documentation as appendix, see also item X26), as this information may have an effect on user self-selection, user expectation and may also bias results.

	1	2	3	4	5	
subitem not at all important	( )	(•)	( )	( )	( )	essential

Does your paper address subitem 4a-iii?

Copy and paste relevant sections from the manuscript (include quotes in quotation marks "like this" to indicate direct quotes from your manuscript), or elaborate on this item by providing additional information not in the ms, or briefly explain why the item is not applicable/relevant for your study

"Participants will be recruited within each settlement using purposive methods, including word-of-mouth and venue-based sampling at refugee agencies and community events. Priority will given to participants that participated in previous trials with the same research group on HIV self-testing and COVID-19 [45]. There will be additional purposive recruitment of 16 and 17 year old participants in order to renew the cohort. Peer navigators will facilitate participant retention and maintain engagement in the study using multiple study reminder strategies including SMS reminders and visits. We will utilize existing outreach and services by local refugee agencies and community partners."
"Peer navigators will facilitate participant retention and maintain engagement in the study using multiple study reminder strategies including SMS reminders and visits. We will utilize existing outreach and services by local refugee agencies and community partners."

4b) Settings and locations where the data were collected

Does your paper address CONSORT subitem 4b? *

Copy and paste relevant sections from the manuscript (include quotes in quotation marks "like this" to indicate direct quotes from your manuscript), or elaborate on this item by providing additional information not in the ms, or briefly explain why the item is not applicable/relevant for your study

"The five informal settlements selected for this study are grouped into three sites based on their proximity to one another (Kabalagala/Kansanga, Katwe/Nsambya, and Rubaga) and have been purposively chosen because these communities host many displaced/refugee persons in Kampala"

4b-i) Report if outcomes were (self-)assessed through online questionnaires

Clearly report if outcomes were (self-)assessed through online questionnaires (as common in web-based trials) or otherwise.

	1	2	3	4	5	
subitem not at all important	◉	○	○	○	○	essential

Does your paper address subitem 4b-i? *

Copy and paste relevant sections from the manuscript (include quotes in quotation marks "like this" to indicate direct quotes from your manuscript), or elaborate on this item by providing additional information not in the ms, or briefly explain why the item is not applicable/relevant for your study

"Data will be collected using a mobile-based structured survey on cell phones or tablets in all study languages by trained research assistants. We will collect data using SurveyCTO application (Dobility, Cambridge, the USA), a secure platform that automatically encrypts data, uploading it using a Secure Sockets Layer (SSL) certificate to a password-protected server. SurveyCTO allows for offline data collection, facilitates multi-lingual data collection and has branching logic and consistency checks."

4b-ii) Report how institutional affiliations are displayed

Report how institutional affiliations are displayed to potential participants [on ehealth media], as affiliations with prestigious hospitals or universities may affect volunteer rates, use, and reactions with regards to an intervention.(Not a required item – describe only if this may bias results)

	1	2	3	4	5	
subitem not at all important	(•)	( )	( )	( )	( )	essential

Does your paper address subitem 4b-ii?

Copy and paste relevant sections from the manuscript (include quotes in quotation marks "like this" to indicate direct quotes from your manuscript), or elaborate on this item by providing additional information not in the ms, or briefly explain why the item is not applicable/relevant for your study

No, not applicable.

5) The interventions for each group with sufficient details to allow replication, including how and when they were actually administered

5-i) Mention names, credential, affiliations of the developers, sponsors, and owners

Mention names, credential, affiliations of the developers, sponsors, and owners [6] (if authors/evaluators are owners or developer of the software, this needs to be declared in a “Conflict of interest” section or mentioned elsewhere in the manuscript).

	1	2	3	4	5	
subitem not at all important	( )	(•)	( )	( )	( )	essential

Does your paper address subitem 5-i?

Copy and paste relevant sections from the manuscript (include quotes in quotation marks "like this" to indicate direct quotes from your manuscript), or elaborate on this item by providing additional information not in the ms, or briefly explain why the item is not applicable/relevant for your study

Yes. It is included in the conflict of interest statement.

5-ii) Describe the history/development process

Describe the history/development process of the application and previous formative evaluations (e.g., focus groups, usability testing), as these will have an impact on adoption/use rates and help with interpreting results.

	1	2	3	4	5	
subitem not at all important	( )	( )	( )	(•)	( )	essential

Does your paper address subitem 5-ii?

Copy and paste relevant sections from the manuscript (include quotes in quotation marks "like this" to indicate direct quotes from your manuscript), or elaborate on this item by providing additional information not in the ms, or briefly explain why the item is not applicable/relevant for your study

"The study protocol was developed after a formative qualitative research phase (Phase 1), which included: (i) in-depth semi-structured key informant interviews with professionals in various roles supporting health and well-being of refugee youth in Uganda (n=10); and (ii) age- and-gender-segregated focus groups (n=4 FGDs with 6 people in each focus group; n=24 participants in total) with refugee youth in Kampala aged 16-24 years. One FGD was held with young women ages 16-19, one with young women ages 20-24, one with young men ages 16-19, one with young men ages 20-24). This formative qualitative work explored community perspectives on mental health, mental health literacy and mental health stigma. The qualitative findings were used to identify key themes for the development of the VR scenario, mHealth SMS content and to adapt GPM+ to context and population group. In this way, the study responds to the mental health needs and priorities of refugee youth in this humanitarian context. Further, peer navigators, themselves urban refugee youth living in Kampala's informal settlements, provided feedback for the study design and outcomes and pilot tested the survey."

5-iii) Revisions and updating

Revisions and updating. Clearly mention the date and/or version number of the application/intervention (and comparator, if applicable) evaluated, or describe whether the intervention underwent major changes during the evaluation process, or whether the development and/or content was "frozen" during the trial. Describe dynamic components such as news feeds or changing content which may have an impact on the replicability of the intervention (for unexpected events see item 3b).

	1	2	3	4	5	
subitem not at all important	(•)	( )	( )	( )	( )	essential

Does your paper address subitem 5-iii?

Copy and paste relevant sections from the manuscript (include quotes in quotation marks "like this" to indicate direct quotes from your manuscript), or elaborate on this item by providing additional information not in the ms, or briefly explain why the item is not applicable/relevant for your study

no: this is a protocol paper, not applicable

5-iv) Quality assurance methods

Provide information on quality assurance methods to ensure accuracy and quality of information provided [1], if applicable.

	1	2	3	4	5	
subitem not at all important	( )	(•)	( )	( )	( )	essential

Does your paper address subitem 5-iv?

Copy and paste relevant sections from the manuscript (include quotes in quotation marks "like this" to indicate direct quotes from your manuscript), or elaborate on this item by providing additional information not in the ms, or briefly explain why the item is not applicable/relevant for your study

"Irrespective of study findings, trial results will be published in peer-reviewed scientific journals following international authorship guidelines, and will be presented to academics and researchers at key scientific conferences. Findings will disseminated through a variety of methods to a range of stakeholders, including: (1) academics and researchers in mental health; (2) international collaborating organisations such as WHO and UNHCR; (3) to local the Ugandan Ministries of Health and Education; (4) implementing partners in adolescent health and social work; and (5) community organizations and members. Community reports and policy briefs will be disseminated to the Ugandan Ministries of Health and Education, collaborators [e.g., YARID] and community organisations."

5-v) Ensure replicability by publishing the source code, and/or providing screenshots/screen-capture video, and/or providing flowcharts of the algorithms used

Ensure replicability by publishing the source code, and/or providing screenshots/screen-capture video, and/or providing flowcharts of the algorithms used. Replicability (i.e., other researchers should in principle be able to replicate the study) is a hallmark of scientific reporting.

	1	2	3	4	5	
subitem not at all important	(•)	( )	( )	( )	( )	essential

Does your paper address subitem 5-v?

Copy and paste relevant sections from the manuscript (include quotes in quotation marks "like this" to indicate direct quotes from your manuscript), or elaborate on this item by providing additional information not in the ms, or briefly explain why the item is not applicable/relevant for your study

no: not applicable, this is a protocol

5-vi) Digital preservation

Digital preservation: Provide the URL of the application, but as the intervention is likely to change or disappear over the course of the years; also make sure the intervention is archived (Internet Archive, webcitation.org, and/or publishing the source code or screenshots/videos alongside the article). As pages behind login screens cannot be archived, consider creating demo pages which are accessible without login.

	1	2	3	4	5	
subitem not at all important	(•)	( )	( )	( )	( )	essential

Does your paper address subitem 5-vi?

Copy and paste relevant sections from the manuscript (include quotes in quotation marks "like this" to indicate direct quotes from your manuscript), or elaborate on this item by providing additional information not in the ms, or briefly explain why the item is not applicable/relevant for your study

not applicable, this is a mobile phone application operated via WelTel, not openly available.
the virtual reality app was developed privately and is not available online.

5-vii) Access

Access: Describe how participants accessed the application, in what setting/context, if they had to pay (or were paid) or not, whether they had to be a member of specific group. If known, describe how participants obtained "access to the platform and Internet" [1]. To ensure access for editors/reviewers/readers, consider to provide a "backdoor" login account or demo mode for reviewers/readers to explore the application (also important for archiving purposes, see vi).

	1	2	3	4	5	
subitem not at all important	(•)	( )	( )	( )	( )	essential

Does your paper address subitem 5-vii? *

Copy and paste relevant sections from the manuscript (include quotes in quotation marks "like this" to indicate direct quotes from your manuscript), or elaborate on this item by providing additional information not in the ms, or briefly explain why the item is not applicable/relevant for your study

"Participants in this arm will be enrolled into the Tushirikiane 4-MH intervention, including (1) an immersive, interactive 30-minute virtual reality (VR) session; and, (2) an 8-week web-based short messaging service (SMS) mental health literacy program."
"This platform for this program is hosted by WelTel [48,49], a non-profit agency [50]. WelTel's platform also allows each participant to create their own weekly goals on a secure platform managed by our study team, which will be supported by peer navigators."

5-viii) Mode of delivery, features/functionalities/components of the intervention and comparator, and the theoretical framework

Describe mode of delivery, features/functionalities/components of the intervention and comparator, and the theoretical framework [6] used to design them (instructional strategy [1], behaviour change techniques, persuasive features, etc., see e.g., [7, 8] for terminology). This includes an in-depth description of the content (including where it is coming from and who developed it) [1]," whether [and how] it is tailored to individual circumstances and allows users to track their progress and receive feedback" [6]. This also includes a description of communication delivery channels and – if computer-mediated communication is a component – whether communication was synchronous or asynchronous [6]. It also includes information on presentation strategies [1], including page design principles, average amount of text on pages, presence of hyperlinks to other resources, etc. [1].

	1	2	3	4	5	
subitem not at all important	( )	(•)	( )	( )	( )	essential

Does your paper address subitem 5-viii? *

Copy and paste relevant sections from the manuscript (include quotes in quotation marks "like this" to indicate direct quotes from your manuscript), or elaborate on this item by providing additional information not in the ms, or briefly explain why the item is not applicable/relevant for your study

"Participants in this arm will be enrolled into the Tushirikiane 4-MH intervention (as above) as well as Group Problem Management Plus (GPM+). GPM+ is a World Health Organization brief psychological transdiagnostic intervention for persons experiencing a range of adversities, including poverty and war, that over five group sessions, aims to address both practical (e.g., housing) and emotional (e.g., stress) challenges [24]. Sessions employ evidence-based approaches to stress management, problem solving, behavioural activation, and social support to reduce a range of mental health concerns. The intervention includes five 3-hour sessions, each with a mechanism of action:
(1) Managing stress: mechanism of action-identify goals, learn deep breathing and techniques for stress management;
(2) Managing problems: mechanism of action-identify 1 solvable practical problem, brainstorm possible solutions together;
(3) Get going, keep doing: mechanism of action-learn about depression and inactivity, identify and plan small enjoyable activities;
(4) Strengthen social support: mechanism of action-discuss a range of social support resources, make plan to increase social support; and
(5) Staying well: mechanism of action- reviews all of the mechanisms of action in the prior 4 sessions."
"Participants in this arm will be enrolled into the Tushirikiane 4-MH intervention, including (1) an immersive, interactive 30-minute virtual reality (VR) session; and, (2) an 8-week web-based short messaging service (SMS) mental health literacy program.
The virtual reality scenario was designed based on findings from Phase 1, and includes mental health literacy components [15], alongside components of psychological first aid [46], and self-compassion activities [47]. It was designed to be visibly similar, but non-identifiable within the local context and will be offered in participants' language of choice (French, English, Kirundi, Kinyarwanda or Swahili). Participants will be invited to participate in the VR scenario alone in a private room, or outdoors at the partner organisation. The VR session will be viewed on low-cost VR headsets and study equipment will be sanitized and cleaned between uses. Each youth will receive a brief graphic manual developed by the PN with step-by-step graphic guides to the MHL information and self-compassion exercises to keep with them for practising the activities.
The 8-week mHealth program will include weekly WelTel SMS blasts designed to provide mental health literacy [15], informed psychological first aid [46] and self-compassion activities [47]. It will also include weekly WelTel web-based secure platform social group discussions on MHL, stress coping, and stigma moderated by peer navigators alongside a trained coordinator. Weekly MHL WelTel platform moderated discussion foci will address MHL, stigma and stress coping strategies, including self-compassion and other strategies that emerged from qualitative findings in Phase 1. Diverse methods, including: 'scenarios' mimicking real life situations, a 'question box', sharing photos demonstrating stress coping strategies, memes, songs and ways participants interrupted stigma in their daily lives."

5-ix) Describe use parameters

Describe use parameters (e.g., intended "doses" and optimal timing for use). Clarify what instructions or recommendations were given to the user, e.g., regarding timing, frequency, heaviness of use, if any, or was the intervention used ad libitum.

	1	2	3	4	5	
subitem not at all important	○	◉	○	○	○	essential

Does your paper address subitem 5-ix?

Copy and paste relevant sections from the manuscript (include quotes in quotation marks "like this" to indicate direct quotes from your manuscript), or elaborate on this item by providing additional information not in the ms, or briefly explain why the item is not applicable/relevant for your study

"Participants in this arm will be enrolled into the Tushirikiane 4-MH intervention (as above) as well as Group Problem Management Plus (GPM+). GPM+ is a World Health Organization brief psychological transdiagnostic intervention for persons experiencing a range of adversities, including poverty and war, that over five group sessions, aims to address both practical (e.g., housing) and emotional (e.g., stress) challenges [24]. Sessions employ evidence-based approaches to stress management, problem solving, behavioural activation, and social support to reduce a range of mental health concerns. The intervention includes five 3-hour sessions, each with a mechanism of action:
(1) Managing stress: mechanism of action-identify goals, learn deep breathing and techniques for stress management;
(2) Managing problems: mechanism of action-identify 1 solvable practical problem, brainstorm possible solutions together;
(3) Get going, keep doing: mechanism of action-learn about depression and inactivity, identify and plan small enjoyable activities;
(4) Strengthen social support: mechanism of action-discuss a range of social support resources, make plan to increase social support; and
(5) Staying well: mechanism of action- reviews all of the mechanisms of action in the prior 4 sessions."
"Participants in this arm will be enrolled into the Tushirikiane 4-MH intervention, including (1) an immersive, interactive 30-minute virtual reality (VR) session; and, (2) an 8-week web-based short messaging service (SMS) mental health literacy program.
The virtual reality scenario was designed based on findings from Phase 1, and includes mental health literacy components [15], alongside components of psychological first aid [46], and self-compassion activities [47]. It was designed to be visibly similar, but non-identifiable within the local context and will be offered in participants' language of choice (French, English, Kirundi, Kinyarwanda or Swahili). Participants will be invited to participate in the VR scenario alone in a private room, or outdoors at the partner organisation. The VR session will be viewed on low-cost VR headsets and study equipment will be sanitized and cleaned between uses. Each youth will receive a brief graphic manual developed by the PN with step-by-step graphic guides to the MHL information and self-compassion exercises to keep with them for practising the activities.
The 8-week mHealth program will include weekly WelTel SMS blasts designed to provide mental health literacy [15], informed psychological first aid [46] and self-compassion activities [47]. It will also include weekly WelTel web-based secure platform social group discussions on MHL, stress coping, and stigma moderated by peer navigators alongside a trained coordinator. Weekly MHL WelTel platform moderated discussion foci will address MHL, stigma and stress coping strategies, including self-compassion and other strategies that emerged from qualitative findings in Phase 1. Diverse methods, including: 'scenarios' mimicking real life situations, a 'question box', sharing photos demonstrating stress coping strategies, memes, songs and ways participants interrupted stigma in their daily lives."

5-x) Clarify the level of human involvement

Clarify the level of human involvement (care providers or health professionals, also technical assistance) in the e-intervention or as co-intervention (detail number and expertise of professionals involved, if any, as well as "type of assistance offered, the timing and frequency of the support, how it is initiated, and the medium by which the assistance is delivered". It may be necessary to distinguish between the level of human involvement required for the trial, and the level of human involvement required for a routine application outside of a RCT setting (discuss under item 21 – generalizability).

	1	2	3	4	5	
subitem not at all important	○	○	◉	○	○	essential

Does your paper address subitem 5-x?

Copy and paste relevant sections from the manuscript (include quotes in quotation marks "like this" to indicate direct quotes from your manuscript), or elaborate on this item by providing additional information not in the ms, or briefly explain why the item is not applicable/relevant for your study

"Participants in this arm will be enrolled into the Tushirikiane 4-MH intervention (as above) as well as Group Problem Management Plus (GPM+). GPM+ is a World Health Organization brief psychological transdiagnostic intervention for persons experiencing a range of adversities, including poverty and war, that over five group sessions, aims to address both practical (e.g., housing) and emotional (e.g., stress) challenges [24]. Sessions employ evidence-based approaches to stress management, problem solving, behavioural activation, and social support to reduce a range of mental health concerns. The intervention includes five 3-hour sessions, each with a mechanism of action:
(1) Managing stress: mechanism of action-identify goals, learn deep breathing and techniques for stress management;
(2) Managing problems: mechanism of action-identify 1 solvable practical problem, brainstorm possible solutions together;
(3) Get going, keep doing: mechanism of action-learn about depression and inactivity, identify and plan small enjoyable activities;
(4) Strengthen social support: mechanism of action-discuss a range of social support resources, make plan to increase social support; and
(5) Staying well: mechanism of action- reviews all of the mechanisms of action in the prior 4 sessions."
"Participants in this arm will be enrolled into the Tushirikiane 4-MH intervention, including (1) an immersive, interactive 30-minute virtual reality (VR) session; and, (2) an 8-week web-based short messaging service (SMS) mental health literacy program.
The virtual reality scenario was designed based on findings from Phase 1, and includes mental health literacy components [15], alongside components of psychological first aid [46], and self-compassion activities [47]. It was designed to be visibly similar, but non-identifiable within the local context and will be offered in participants' language of choice (French, English, Kirundi, Kinyarwanda or Swahili). Participants will be invited to participate in the VR scenario alone in a private room, or outdoors at the partner organisation. The VR session will be viewed on low-cost VR headsets and study equipment will be sanitized and cleaned between uses. Each youth will receive a brief graphic manual developed by the PN with step-by-step graphic guides to the MHL information and self-compassion exercises to keep with them for practising the activities.
The 8-week mHealth program will include weekly WelTel SMS blasts designed to provide mental health literacy [15], informed psychological first aid [46] and self-compassion activities [47]. It will also include weekly WelTel web-based secure platform social group discussions on MHL, stress coping, and stigma moderated by peer navigators alongside a trained coordinator. Weekly MHL WelTel platform moderated discussion foci will address MHL, stigma and stress coping strategies, including self-compassion and other strategies that emerged from qualitative findings in Phase 1. Diverse methods, including: 'scenarios' mimicking real life situations, a 'question box', sharing photos demonstrating stress coping strategies, memes, songs and ways participants interrupted stigma in their daily lives."

5-xi) Report any prompts/reminders used

Report any prompts/reminders used: Clarify if there were prompts (letters, emails, phone calls, SMS) to use the application, what triggered them, frequency etc. It may be necessary to distinguish between the level of prompts/reminders required for the trial, and the level of prompts/reminders for a routine application outside of a RCT setting (discuss under item 21 – generalizability).

	1	2	3	4	5	
subitem not at all important	○	◉	○	○	○	essential

Does your paper address subitem 5-xi? *

Copy and paste relevant sections from the manuscript (include quotes in quotation marks "like this" to indicate direct quotes from your manuscript), or elaborate on this item by providing additional information not in the ms, or briefly explain why the item is not applicable/relevant for your study

"Participants in this arm will be enrolled into the Tushirikiane 4-MH intervention (as above) as well as Group Problem Management Plus (GPM+). GPM+ is a World Health Organization brief psychological transdiagnostic intervention for persons experiencing a range of adversities, including poverty and war, that over five group sessions, aims to address both practical (e.g., housing) and emotional (e.g., stress) challenges [24]. Sessions employ evidence-based approaches to stress management, problem solving, behavioural activation, and social support to reduce a range of mental health concerns. The intervention includes five 3-hour sessions, each with a mechanism of action:
(1) Managing stress: mechanism of action-identify goals, learn deep breathing and techniques for stress management;
(2) Managing problems: mechanism of action-identify 1 solvable practical problem, brainstorm possible solutions together;
(3) Get going, keep doing: mechanism of action-learn about depression and inactivity, identify and plan small enjoyable activities;
(4) Strengthen social support: mechanism of action-discuss a range of social support resources, make plan to increase social support; and
(5) Staying well: mechanism of action- reviews all of the mechanisms of action in the prior 4 sessions."
"Participants in this arm will be enrolled into the Tushirikiane 4-MH intervention, including (1) an immersive, interactive 30-minute virtual reality (VR) session; and, (2) an 8-week web-based short messaging service (SMS) mental health literacy program.
The virtual reality scenario was designed based on findings from Phase 1, and includes mental health literacy components [15], alongside components of psychological first aid [46], and self-compassion activities [47]. It was designed to be visibly similar, but non-identifiable within the local context and will be offered in participants' language of choice (French, English, Kirundi, Kinyarwanda or Swahili). Participants will be invited to participate in the VR scenario alone in a private room, or outdoors at the partner organisation. The VR session will be viewed on low-cost VR headsets and study equipment will be sanitized and cleaned between uses. Each youth will receive a brief graphic manual developed by the PN with step-by-step graphic guides to the MHL information and self-compassion exercises to keep with them for practising the activities.
The 8-week mHealth program will include weekly WelTel SMS blasts designed to provide mental health literacy [15], informed psychological first aid [46] and self-compassion activities [47]. It will also include weekly WelTel web-based secure platform social group discussions on MHL, stress coping, and stigma moderated by peer navigators alongside a trained coordinator. Weekly MHL WelTel platform moderated discussion foci will address MHL, stigma and stress coping strategies, including self-compassion and other strategies that emerged from qualitative findings in Phase 1. Diverse methods, including: 'scenarios' mimicking real life situations, a 'question box', sharing photos demonstrating stress coping strategies, memes, songs and ways participants interrupted stigma in their daily lives."

5-xii) Describe any co-interventions (incl. training/support)

Describe any co-interventions (incl. training/support): Clearly state any interventions that are provided in addition to the targeted eHealth intervention, as ehealth intervention may not be designed as stand-alone intervention. This includes training sessions and support [1]. It may be necessary to distinguish between the level of training required for the trial, and the level of training for a routine application outside of a RCT setting (discuss under item 21 – generalizability.

	1	2	3	4	5	
subitem not at all important	○	◉	○	○	○	essential

Does your paper address subitem 5-xii? *

Copy and paste relevant sections from the manuscript (include quotes in quotation marks "like this" to indicate direct quotes from your manuscript), or elaborate on this item by providing additional information not in the ms, or briefly explain why the item is not applicable/relevant for your study

"Participants in this arm will be enrolled into the Tushirikiane 4-MH intervention (as above) as well as Group Problem Management Plus (GPM+). GPM+ is a World Health Organization brief psychological transdiagnostic intervention for persons experiencing a range of adversities, including poverty and war, that over five group sessions, aims to address both practical (e.g., housing) and emotional (e.g., stress) challenges [24]. Sessions employ evidence-based approaches to stress management, problem solving, behavioural activation, and social support to reduce a range of mental health concerns. The intervention includes five 3-hour sessions, each with a mechanism of action:
(1) Managing stress: mechanism of action-identify goals, learn deep breathing and techniques for stress management;
(2) Managing problems: mechanism of action-identify 1 solvable practical problem, brainstorm possible solutions together;
(3) Get going, keep doing: mechanism of action-learn about depression and inactivity, identify and plan small enjoyable activities;
(4) Strengthen social support: mechanism of action-discuss a range of social support resources, make plan to increase social support; and
(5) Staying well: mechanism of action- reviews all of the mechanisms of action in the prior 4 sessions."
"Participants in this arm will be enrolled into the Tushirikiane 4-MH intervention, including (1) an immersive, interactive 30-minute virtual reality (VR) session; and, (2) an 8-week web-based short messaging service (SMS) mental health literacy program.
The virtual reality scenario was designed based on findings from Phase 1, and includes mental health literacy components [15], alongside components of psychological first aid [46], and self-compassion activities [47]. It was designed to be visibly similar, but non-identifiable within the local context and will be offered in participants' language of choice (French, English, Kirundi, Kinyarwanda or Swahili). Participants will be invited to participate in the VR scenario alone in a private room, or outdoors at the partner organisation. The VR session will be viewed on low-cost VR headsets and study equipment will be sanitized and cleaned between uses. Each youth will receive a brief graphic manual developed by the PN with step-by-step graphic guides to the MHL information and self-compassion exercises to keep with them for practising the activities.
The 8-week mHealth program will include weekly WelTel SMS blasts designed to provide mental health literacy [15], informed psychological first aid [46] and self-compassion activities [47]. It will also include weekly WelTel web-based secure platform social group discussions on MHL, stress coping, and stigma moderated by peer navigators alongside a trained coordinator. Weekly MHL WelTel platform moderated discussion foci will address MHL, stigma and stress coping strategies, including self-compassion and other strategies that emerged from qualitative findings in Phase 1. Diverse methods, including: 'scenarios' mimicking real life situations, a 'question box', sharing photos demonstrating stress coping strategies, memes, songs and ways participants interrupted stigma in their daily lives."
"Adverse events can also be directly reported by study participants via a Tushirikiane-4MH toll-free hotline, which will be shared with the study participant at enrollment and monitored by trained counselors throughout the duration of the study. Any adverse event requiring a narrative form will be reported to the principal investigators within 24 hours."

6a) Completely defined pre-specified primary and secondary outcome measures, including how and when they were assessed

Does your paper address CONSORT subitem 6a? *

Copy and paste relevant sections from the manuscript (include quotes in quotation marks "like this" to indicate direct quotes from your manuscript), or elaborate on this item by providing additional information not in the ms, or briefly explain why the item is not applicable/relevant for your study

"This study aims to evaluate the feasibility and effectiveness of youth-tailored, mHealth-supported mental health interventions among refugee and displaced youth aged 16-24 years in Kampala, Uganda. It will evaluate two strategies: a virtual reality experience, and an adapted version of the WHO's GPM+. Both interventions will supported by mobile health (mHealth) SMS check-ins."
"Primary outcomes (mental health literacy, attitudes towards mental help-seeking, depression, adaptive coping, mental health stigma, mental wellbeing, level of functioning) and secondary outcomes of intervention feasibility will be evaluated."

6a-i) Online questionnaires: describe if they were validated for online use and apply CHERRIES items to describe how the questionnaires were designed/deployed

If outcomes were obtained through online questionnaires, describe if they were validated for online use and apply CHERRIES items to describe how the questionnaires were designed/deployed [9].

	1	2	3	4	5	
subitem not at all important	◉	○	○	○	○	essential

Does your paper address subitem 6a-i?

Copy and paste relevant sections from manuscript text

no: not applicable, they are interviewer administered and not online questionnaires

6a-ii) Describe whether and how "use" (including intensity of use/dosage) was defined/measured/monitored

Describe whether and how "use" (including intensity of use/dosage) was defined/measured/monitored (logins, logfile analysis, etc.). Use/adoption metrics are important process outcomes that should be reported in any ehealth trial.

	1	2	3	4	5

subitem not at all important ( ) (•) ( ) ( ) ( ) essential

Does your paper address subitem 6a-ii?

Copy and paste relevant sections from manuscript text

yes: The WelTel system will manage the SMS intervention on a
structured mobile phone platform (all SMS interactions are logged).

6a-iii) Describe whether, how, and when qualitative feedback from participants was obtained

Describe whether, how, and when qualitative feedback from participants was obtained (e.g., through emails, feedback forms, interviews, focus groups).

	1	2	3	4	5	
subitem not at all important	( )	( )	( )	(•)	( )	essential

Does your paper address subitem 6a-iii?

Copy and paste relevant sections from manuscript text

"The study protocol was developed after a formative qualitative research phase (Phase 1), which included: (i) in-depth semi-structured key informant interviews with professionals in various roles supporting health and well-being of refugee youth in Uganda (n=10); and (ii) age- and-gender-segregated focus groups (n=4 FGDs with 6 people in each focus group; n=24 participants in total) with refugee youth in Kampala aged 16-24 years. One FGD was held with young women ages 16-19, one with young women ages 20-24, one with young men ages 16-19, one with young men ages 20-24). This formative qualitative work explored community perspectives on mental health, mental health literacy and mental health stigma. The qualitative findings were used to identify key themes for the development of the VR scenario, mHealth SMS content and to adapt GPM+ to context and population group. In this way, the study responds to the mental health needs and priorities of refugee youth in this humanitarian context. Further, peer navigators, themselves urban refugee youth living in Kampala's informal settlements, provided feedback for the study design and outcomes and pilot tested the survey."

6b) Any changes to trial outcomes after the trial commenced, with reasons

Does your paper address CONSORT subitem 6b? *

Copy and paste relevant sections from the manuscript (include quotes in quotation marks "like this" to indicate direct quotes from your manuscript), or elaborate on this item by providing additional information not in the ms, or briefly explain why the item is not applicable/relevant for your study

There were no changes to the trial outcomes.

7a) How sample size was determined

NPT: When applicable, details of whether and how the clustering by care provides or centers was addressed

7a-i) Describe whether and how expected attrition was taken into account when calculating the sample size

Describe whether and how expected attrition was taken into account when calculating the sample size.

	1	2	3	4	5	
subitem not at all important	○	○	⦿	○	○	essential

Does your paper address subitem 7a-i?

Copy and paste relevant sections from manuscript title (include quotes in quotation marks "like this" to indicate direct quotes from your manuscript), or elaborate on this item by providing additional information not in the ms, or briefly explain why the item is not applicable/relevant for your study

"Cluster sizes of 90 per group (n=270) are required to have 80% power (p<0.05) to detect a difference of ~3 points in mean depression score (moderate effect size), at a level of significance of ±=0.05, assuming an intraclass correlation of 0.01 and standard deviation of 7. This design will allow us to produce evidence of mental health benefits of a combination of approaches: first approach is VR + MHL alone; 2nd approach is VR + MHL alongside Group PM+; and the third approach, with the waitlist control, will test effects of Group PM+ alone With 10% attrition, 297 participants (99 per cluster) are required."

7b) When applicable, explanation of any interim analyses and stopping guidelines

Does your paper address CONSORT subitem 7b? *

Copy and paste relevant sections from the manuscript (include quotes in quotation marks "like this" to indicate direct quotes from your manuscript), or elaborate on this item by providing additional information not in the ms, or briefly explain why the item is not applicable/relevant for your study

no: not applicable, this is a protocol paper.

8a) Method used to generate the random allocation sequence

NPT: When applicable, how care providers were allocated to each trial group

Does your paper address CONSORT subitem 8a? *

Copy and paste relevant sections from the manuscript (include quotes in quotation marks "like this" to indicate direct quotes from your manuscript), or elaborate on this item by providing additional information not in the ms, or briefly explain why the item is not applicable/relevant for your study

yes: The study has been designed as a three-arm cRCT consisting
of two treatment arms and a waitlist arm. Clusters will be randomized to one of the following three arms: (1) virtual reality + mHealth mental health literacy; (2) the above + group problem management plus (GPM+) and (3) waitlist, followed by GPM+.

8b) Type of randomisation; details of any restriction (such as blocking and block size)

Does your paper address CONSORT subitem 8b? *

Copy and paste relevant sections from the manuscript (include quotes in quotation marks "like this" to indicate direct quotes from your manuscript), or elaborate on this item by providing additional information not in the ms, or briefly explain why the item is not applicable/relevant for your study

"To evaluate intervention effectiveness, we will conduct a cluster randomized controlled trial (cRCT). The clusters include five informal settlements grouped into three sites that are randomized in a 1:1:1 method to one of three study arms. Once the youth have provided informed written consent they will be enrolled into the study and assigned to a study arm based on the community they live in. Although outcome data will be collected at the level of the individual, we selected cluster randomization over individual randomization because the intervention is implemented at the settlement level. A cluster randomized design addresses threats of internal validity. It reduces the possibility of experimental contamination due to the shared social and physical environments between youth in the same or nearby informal settlements [40]. Data will be collected at three time points: baseline enrolment into the intervention, at eight-weeks after implementation, and sixteen-weeks after implementation."

9) Mechanism used to implement the random allocation sequence (such as sequentially numbered containers), describing any steps taken to conceal the sequence until interventions were assigned

Does your paper address CONSORT subitem 9? *

Copy and paste relevant sections from the manuscript (include quotes in quotation marks "like this" to indicate direct quotes from your manuscript), or elaborate on this item by providing additional information not in the ms, or briefly explain why the item is not applicable/relevant for your study

No: not applicable, not blinded

10) Who generated the random allocation sequence, who enrolled participants, and who assigned participants to interventions

Does your paper address CONSORT subitem 10? *

Copy and paste relevant sections from the manuscript (include quotes in quotation marks "like this" to indicate direct quotes from your manuscript), or elaborate on this item by providing additional information not in the ms, or briefly explain why the item is not applicable/relevant for your study

"The study has been designed as a three-arm cRCT consisting of two treatment arms and one control arm. Clusters will be randomized to one of three arms: (1) virtual reality (VR) + SMS mental health literacy (MHL); (2) VR + MHL + Group Problem Management Plus (GPM+); or, (3) waitlist, followed by GPM+ after intervention implementation and follow-up. Data will be collected a baseline, after intervention implementation (8 weeks) and at a follow-up visit (at 16 weeks). The third arm will receive an additional survey an additional 8 weeks after to see changes after participating in GPM+. All study members wil receive YARID resources and referrals as needed for mental health support from a trained social worker with lived experience as a refugee and fluent in participant languages.
The trial arms and interventions are described below and summarized in Figure 1. (Figure 1)"

11a) If done, who was blinded after assignment to interventions (for example, participants, care providers, those assessing outcomes) and how

NPT: Whether or not administering co-interventions were blinded to group assignment

11a-i) Specify who was blinded, and who wasn’t

Specify who was blinded, and who wasn’t. Usually, in web-based trials it is not possible to blind the participants [1, 3] (this should be clearly acknowledged), but it may be possible to blind outcome assessors, those doing data analysis or those administering co-interventions (if any).

	1	2	3	4	5	
subitem not at all important	(•)	( )	( )	( )	( )	essential

Does your paper address subitem 11a-i? *

Copy and paste relevant sections from the manuscript (include quotes in quotation marks "like this" to indicate direct quotes from your manuscript), or elaborate on this item by providing additional information not in the ms, or briefly explain why the item is not applicable/relevant for your study

not applicable, no blinding

11a-ii) Discuss e.g., whether participants knew which intervention was the “intervention of interest” and which one was the “comparator”

Informed consent procedures (4a-ii) can create biases and certain expectations - discuss e.g., whether participants knew which intervention was the “intervention of interest” and which one was the “comparator”.

	1	2	3	4	5	
subitem not at all important	(•)	( )	( )	( )	( )	essential

Does your paper address subitem 11a-ii?

Copy and paste relevant sections from the manuscript (include quotes in quotation marks "like this" to indicate direct quotes from your manuscript), or elaborate on this item by providing additional information not in the ms, or briefly explain why the item is not applicable/relevant for your study

No: not applicable, not blinded

11b) If relevant, description of the similarity of interventions

(this item is usually not relevant for ehealth trials as it refers to similarity of a placebo or sham intervention to a active medication/intervention)

Does your paper address CONSORT subitem 11b? *

Copy and paste relevant sections from the manuscript (include quotes in quotation marks "like this" to indicate direct quotes from your manuscript), or elaborate on this item by providing additional information not in the ms, or briefly explain why the item is not applicable/relevant for your study

Not applicable

12a) Statistical methods used to compare groups for primary and secondary outcomes

NPT: When applicable, details of whether and how the clustering by care providers or centers was addressed

Does your paper address CONSORT subitem 12a? *

Copy and paste relevant sections from the manuscript (include quotes in quotation marks "like this" to indicate direct quotes from your manuscript), or elaborate on this item by providing additional information not in the ms, or briefly explain why the item is not applicable/relevant for your study

"Analysis and reporting for the cluster-randomized trial (Phase 2) will be conducted in accordance with CONSORT (Consolidated Standards of Reporting Trials) guidelines [54] (Multimedia Appendix 1). The analyst will be blinded to group allocation. A flow diagram will be used to illustrate patient flow (screening, randomization, allocation, follow-up). Baseline data will be reported for all three groups and summarised as mean (standard deviation) or median (first quartile, third quartile) for continuous variables and counts and number (percent) for categorical variables. Primary analysis will be by intention-to-treat analysis (data from participants will be analysed according to their allocation, irrespective of whether they actually received that intervention).

We will conduct multivariable regression analyses, adjusting for the outcome measure at baseline and stratification variables. For the primary analysis to assess differences between the three intervention conditions on the outcomes (MHL, attitudes towards MH help-seeking, depression, adaptive coping, mental health stigma, mental wellbeing, level of functioning), indicator variables include intervention assignment, and a vector of baseline covariates (e.g., socio-demographics). Analyses will adjust for multiple comparisons across three intervention conditions by using Fisher's protected least significant difference, first assessing differences between intervention groups and if the Omnibus F test is significant, then calculating pairwise comparisons.

Between group comparisons will be done using multi-level mixed effect logistic or linear regression models (to account for clustering) depending on which outcome is being evaluated. For these models, the intervention group will be entered as a fixed effect. The significance level will be set at alpha=0.05. The results will be expressed as odds ratios or mean differences as appropriate, accompanied by 95% confidence intervals and P values.

We will conduct an adjusted analysis for the primary outcome (MHL, attitudes towards MH help-seeking, depression, adaptive coping, mental health stigma, mental wellbeing, level of functioning) to investigate the role of various covariates in the relative effect. Covariates (e.g. age) will be entered as a block. We will explore gender differences in primary and secondary intervention outcomes."

12a-i) Imputation techniques to deal with attrition / missing values

Imputation techniques to deal with attrition / missing values: Not all participants will use the intervention/comparator as intended and attrition is typically high in ehealth trials. Specify how participants who did not use the application or dropped out from the trial were treated in the statistical analysis (a complete case analysis is strongly discouraged, and

simple imputation techniques such as LOCF may also be problematic [4]).

	1	2	3	4	5	
subitem not at all important	◉	○	○	○	○	essential

Does your paper address subitem 12a-i? *

Copy and paste relevant sections from the manuscript (include quotes in quotation marks "like this" to indicate direct quotes from your manuscript), or elaborate on this item by providing additional information not in the ms, or briefly explain why the item is not applicable/relevant for your study

No. Not applicable.

12b) Methods for additional analyses, such as subgroup analyses and adjusted analyses

Does your paper address CONSORT subitem 12b? *

Copy and paste relevant sections from the manuscript (include quotes in quotation marks "like this" to indicate direct quotes from your manuscript), or elaborate on this item by providing additional information not in the ms, or briefly explain why the item is not applicable/relevant for your study

yes: Covariates (eg, age) will be entered as a block. We will explore gender differences in primary and secondary intervention outcomes.

X26) REB/IRB Approval and Ethical Considerations [recommended as subheading under "Methods"] (not a CONSORT item)

X26-i) Comment on ethics committee approval

	1	2	3	4	5	
subitem not at all important	○	○	○	◉	○	essential

Does your paper address subitem X26-i?

Copy and paste relevant sections from the manuscript (include quotes in quotation marks "like this" to indicate direct quotes from your manuscript), or elaborate on this item by providing additional information not in the ms, or briefly

explain why the item is not applicable/relevant for your study

The Tushirikiane4MH trial protocol has been approved by the Research Ethics Boards University of Toronto (12 May 2021), Mildmay Uganda Research Ethics Committee (MUREC) (24 June 2021) and Uganda National Council for Science & Technology (UNCST).

x26-ii) Outline informed consent procedures

Outline informed consent procedures e.g., if consent was obtained offline or online (how? Checkbox, etc.?), and what information was provided (see 4a-ii). See [6] for some items to be included in informed consent documents.

	1	2	3	4	5	
subitem not at all important	○	○	⊙	○	○	essential

Does your paper address subitem X26-ii?

Copy and paste relevant sections from the manuscript (include quotes in quotation marks "like this" to indicate direct quotes from your manuscript), or elaborate on this item by providing additional information not in the ms, or briefly explain why the item is not applicable/relevant for your study

"The protocol for the study was developed in accordance with the SPIRIT Statement [55,56]. The study population includes young adults (aged 16 and over) capable of providing informed consent. We received ethics approval to allow youth aged 16-17 years to participate without parental consent; this is a common approach to reduce barriers to youth participation in health research on potentially sensitive topics [57,58].
All participants will receive information about the study before being enrolled to ensure understanding of rights for refusal/withdrawal, study processes, and expectations and provide written informed consent. To ensure the protection of human subjects, all participants in will be provided with enough time to provide written voluntary consent to participate in the study. All informed written consent processes will occur in a private room at a location provided by YARID. The participant will read the consent form themselves or a peer navigator will read aloud the informed consent in a language comfortable to the participant (French, English, Luganda, Kirundi, Kinyarwanda or Swahili) and will ask if the participant has any questions and will answer their questions. Participants will be asked to sign the consent form or provide a thumbprint to indicate their consent. The consent form will in no way be connected with data collected and will be destroyed five years after data collection is completed.
At any time during the study data collection period, participants can withdraw from the study before completing the interview with no adverse consequences on the care or services they receive. All data will be stored on password-protected computers. To maintain confidentiality, all participants will be given a unique Case ID, and no personal identifying information will be stored with the study data."

X26-iii) Safety and security procedures

Safety and security procedures, incl. privacy considerations, and any steps taken to reduce the likelihood or detection of harm (e.g., education and training, availability of a hotline)

	1	2	3	4	5	
subitem not at all important	○	○	⊙	○	○	essential

Does your paper address subitem X26-iii?

Copy and paste relevant sections from the manuscript (include quotes in quotation marks "like this" to indicate direct quotes from your manuscript), or elaborate on this item by providing additional information not in the ms, or briefly explain why the item is not applicable/relevant for your study

"The risks associated with the Tushirikiane4MH trial are reasonable. All intervention components have all been designed as youth-friendly, based on the principles of psychological first aid and evidence with adult populations. Phase 1 findings informed intervention design, and the interventions have further been piloted with youth peer navigators. Although these interventions are not expected to cause psychological distress, this risk will be shared with participants. Peer navigators have been trained in psychological first aid, and trained counsellors will be on-site throughout the intervention. All participants will also be provided with a list of community resources.
In the case of any adverse event, it will be reported by the peer navigators to the research assistant, who will fill out an adverse event reporting form (Adverse Event Reporting Form), and adverse event narrative form if appropriate (Adverse Event Narrative Form). Adverse events can also be directly reported by study participants via a Tushirikiane-4MH toll-free hotline, which will be shared with the study participant at enrollment and monitored by trained counselors throughout the duration of the study. Any adverse event requiring a narrative form will be reported to the principal investigators within 24 hours."

RESULTS

13a) For each group, the numbers of participants who were randomly assigned, received intended treatment, and were analysed for the primary outcome

NPT: The number of care providers or centers performing the intervention in each group and the number of patients treated by each care provider in each center

Does your paper address CONSORT subitem 13a? *

Copy and paste relevant sections from the manuscript (include quotes in quotation marks "like this" to indicate direct quotes from your manuscript), or elaborate on this item by providing additional information not in the ms, or briefly explain why the item is not applicable/relevant for your study

Not applicable. This is a protocol.

13b) For each group, losses and exclusions after randomisation, together with reasons

Does your paper address CONSORT subitem 13b? (NOTE: Preferably, this is shown in a CONSORT flow diagram) *

Copy and paste relevant sections from the manuscript (include quotes in quotation marks "like this" to indicate direct quotes from your manuscript), or elaborate on this item by providing additional information not in the ms, or briefly explain why the item is not applicable/relevant for your study

No: not applicable, this is a protocol

13b-i) Attrition diagram

Strongly recommended: An attrition diagram (e.g., proportion of participants still logging in or using the intervention/comparator in each group plotted over time, similar to a survival curve) or other figures or tables demonstrating usage/dose/engagement.

	1	2	3	4	5	
subitem not at all important	●	○	○	○	○	essential

Does your paper address subitem 13b-i?

Copy and paste relevant sections from the manuscript or cite the figure number if applicable (include quotes in quotation marks "like this" to indicate direct quotes from your manuscript), or elaborate on this item by providing additional information not in the ms, or briefly explain why the item is not applicable/relevant for your study

no: not applicable, this is a protocol

14a) Dates defining the periods of recruitment and follow-up

Does your paper address CONSORT subitem 14a? *

Copy and paste relevant sections from the manuscript (include quotes in quotation marks "like this" to indicate direct quotes from your manuscript), or elaborate on this item by providing additional information not in the ms, or briefly explain why the item is not applicable/relevant for your study

The formative qualitative phase of Tushirikiane4MH (Supporting Each Other for Mental Health) launched in November, 2021. All qualitative activities from Phase 1 have been completed, as has the development of the VR scenario, WelTel SMS content and adaptation of GPM+. Research staff have been trained. We anticipate for the intervention to begin in June 2022 along with baseline data collection, and the final follow-up survey to be conducted in November 2022.

14a-i) Indicate if critical “secular events” fell into the study period

Indicate if critical “secular events” fell into the study period, e.g., significant changes in Internet resources available or “changes in computer hardware or Internet delivery resources”

	1	2	3	4	5	
subitem not at all important	(•)	( )	( )	( )	( )	essential

Does your paper address subitem 14a-i?

Copy and paste relevant sections from the manuscript (include quotes in quotation marks "like this" to indicate direct quotes from your manuscript), or elaborate on this item by providing additional information not in the ms, or briefly explain why the item is not applicable/relevant for your study

No critical secular events occurred during the study period.

14b) Why the trial ended or was stopped (early)

Does your paper address CONSORT subitem 14b? *

Copy and paste relevant sections from the manuscript (include quotes in quotation marks "like this" to indicate direct quotes from your manuscript), or elaborate on this item by providing additional information not in the ms, or briefly explain why the item is not applicable/relevant for your study

not applicable, this is a protocol

15) A table showing baseline demographic and clinical characteristics for each group

NPT: When applicable, a description of care providers (case volume, qualification, expertise, etc.) and centers (volume) in each group

Does your paper address CONSORT subitem 15? *

Copy and paste relevant sections from the manuscript (include quotes in quotation marks "like this" to indicate direct quotes from your manuscript), or elaborate on this item by providing additional information not in the ms, or briefly explain why the item is not applicable/relevant for your study

no: not applicable, this is a protocol

15-i) Report demographics associated with digital divide issues

In ehealth trials it is particularly important to report demographics associated with digital divide issues, such as age, education, gender, social-economic status, computer/Internet/ehealth literacy of the participants, if known.

	1	2	3	4	5	
subitem not at all important	(•)	( )	( )	( )	( )	essential

Does your paper address subitem 15-i? *

Copy and paste relevant sections from the manuscript (include quotes in quotation marks "like this" to indicate direct quotes from your manuscript), or elaborate on this item by providing additional information not in the ms, or briefly explain why the item is not applicable/relevant for your study

no: not applicable, this is a protocol. As per above we will do analyses testing covariates including age and gender.

16) For each group, number of participants (denominator) included in each analysis and whether the analysis was by original assigned groups

16-i) Report multiple "denominators" and provide definitions

Report multiple "denominators" and provide definitions: Report N's (and effect sizes) "across a range of study participation [and use] thresholds" [1], e.g., N exposed, N consented, N used more than x times, N used more than y weeks, N participants "used" the intervention/comparator at specific pre-defined time points of interest (in absolute and relative numbers per group). Always clearly define "use" of the intervention.

	1	2	3	4	5	
subitem not at all important	○	○	○	○	○	essential

Does your paper address subitem 16-i? *

Copy and paste relevant sections from the manuscript (include quotes in quotation marks "like this" to indicate direct quotes from your manuscript), or elaborate on this item by providing additional information not in the ms, or briefly explain why the item is not applicable/relevant for your study

Groups as assigned were compared on physical activity and quality of life changes, retention, adherence, engagement and satisfaction. However due to the low participation in the coaching sessions, additional analyses were conducted to compare the retention, adherence, engagement and satisfaction of the participants who competed a coaching session to all other participants.

16-ii) Primary analysis should be intent-to-treat

Primary analysis should be intent-to-treat, secondary analyses could include comparing only "users", with the appropriate caveats that this is no longer a randomized sample (see 18-i).

	1	2	3	4	5	
subitem not at all important	◉	○	○	○	○	essential

Does your paper address subitem 16-ii?

Copy and paste relevant sections from the manuscript (include quotes in quotation marks "like this" to indicate direct quotes from your manuscript), or elaborate on this item by providing additional information not in the ms, or briefly explain why the item is not applicable/relevant for your study

Yes: Primary analysis will involve intention-to-treat analysis (data from participants will be analyzed according to their allocation, irrespective of whether they actually received that intervention).

17a) For each primary and secondary outcome, results for each

group, and the estimated effect size and its precision (such as 95% confidence interval)

Does your paper address CONSORT subitem 17a? *

Copy and paste relevant sections from the manuscript (include quotes in quotation marks "like this" to indicate direct quotes from your manuscript), or elaborate on this item by providing additional information not in the ms, or briefly explain why the item is not applicable/relevant for your study

no: not applicable, this is a protocol, but we do include this in the methods: "Between group comparisons will be done using multi-level mixed effect logistic or linear regression models (to account for clustering) depending on which outcome is being evaluated. For these models, the intervention group will be entered as a fixed effect. The significance level will be set at alpha=0.05. The results will be expressed as odds ratios or mean differences as appropriate, accompanied by 95% confidence intervals and P values. We will conduct an adjusted analysis for the primary outcome (MHL, attitudes towards MH help-seeking, depression, adaptive coping, mental health stigma, mental wellbeing, level o functioning) to investigate the role of various covariates in the relative effect. Covariates (e.g. age) will be entered as a block. We will explore gender differences in primary and secondary intervention outcomes."

17a-i) Presentation of process outcomes such as metrics of use and intensity of use

In addition to primary/secondary (clinical) outcomes, the presentation of process outcomes such as metrics of use and intensity of use (dose, exposure) and their operational definitions is critical. This does not only refer to metrics of attrition (13-b) (often a binary variable), but also to more continuous exposure metrics such as "average session length". These must be accompanied by a technical description how a metric like a "session" is defined (e.g., timeout after idle time) [1] (report under item 6a).

	1	2	3	4	5	
subitem not at all important	◉	○	○	○	○	essential

Does your paper address subitem 17a-i?

Copy and paste relevant sections from the manuscript (include quotes in quotation marks "like this" to indicate direct quotes from your manuscript), or elaborate on this item by providing additional information not in the ms, or briefly explain why the item is not applicable/relevant for your study

"no: not applicable, this is a protocol"

17b) For binary outcomes, presentation of both absolute and relative effect sizes is recommended

Does your paper address CONSORT subitem 17b? *

Copy and paste relevant sections from the manuscript (include quotes in quotation marks "like this" to indicate direct quotes from your manuscript), or elaborate on this item by providing additional information not in the ms, or briefly explain why the item is not applicable/relevant for your study

no: not applicable, this is a protocol

18) Results of any other analyses performed, including subgroup analyses and adjusted analyses, distinguishing pre-specified from exploratory

Does your paper address CONSORT subitem 18? *

Copy and paste relevant sections from the manuscript (include quotes in quotation marks "like this" to indicate direct quotes from your manuscript), or elaborate on this item by providing additional information not in the ms, or briefly explain why the item is not applicable/relevant for your study

no: not applicable, this is a protocol

18-i) Subgroup analysis of comparing only users

A subgroup analysis of comparing only users is not uncommon in ehealth trials, but if done, it must be stressed that this is a self-selected sample and no longer an unbiased sample from a randomized trial (see 16-iii).

	1	2	3	4	5	
subitem not at all important	(●)	( )	( )	( )	( )	essential

Does your paper address subitem 18-i?

Copy and paste relevant sections from the manuscript (include quotes in quotation marks "like this" to indicate direct quotes from your manuscript), or elaborate on this item by providing additional information not in the ms, or briefly explain why the item is not applicable/relevant for your study

no: not applicable, this is a protocol

19) All important harms or unintended effects in each group

(for specific guidance see CONSORT for harms)

Does your paper address CONSORT subitem 19? *

Copy and paste relevant sections from the manuscript (include quotes in quotation marks "like this" to indicate direct quotes from your manuscript), or elaborate on this item by providing additional information not in the ms, or briefly explain why the item is not applicable/relevant for your study

no: not applicable, this is a protocol

19-i) Include privacy breaches, technical problems

Include privacy breaches, technical problems. This does not only include physical "harm" to participants, but also incidents such as perceived or real privacy breaches [1], technical problems, and other unexpected/unintended incidents. "Unintended effects" also includes unintended positive effects [2].

	1	2	3	4	5	
subitem not at all important	(•)	( )	( )	( )	( )	essential

Does your paper address subitem 19-i?

Copy and paste relevant sections from the manuscript (include quotes in quotation marks "like this" to indicate direct quotes from your manuscript), or elaborate on this item by providing additional information not in the ms, or briefly explain why the item is not applicable/relevant for your study

no: not applicable, this is a protocol

19-ii) Include qualitative feedback from participants or observations from staff/researchers

Include qualitative feedback from participants or observations from staff/researchers, if available, on strengths and shortcomings of the application, especially if they point to unintended/unexpected effects or uses. This includes (if available) reasons for why people did or did not use the application as intended by the developers.

	1	2	3	4	5	
subitem not at all important	( )	(•)	( )	( )	( )	essential

Does your paper address subitem 19-ii?

Copy and paste relevant sections from the manuscript (include quotes in quotation marks "like this" to indicate direct quotes from your manuscript), or elaborate on this item by providing additional information not in the ms, or briefly explain why the item is not applicable/relevant for your study

no: not applicable, this is a protocol

DISCUSSION

22) Interpretation consistent with results, balancing benefits and harms, and considering other relevant evidence

NPT: In addition, take into account the choice of the comparator, lack of or partial blinding, and unequal expertise of care providers or centers in each group

22-i) Restate study questions and summarize the answers suggested by the data, starting with primary outcomes and process outcomes (use)

Restate study questions and summarize the answers suggested by the data, starting with primary outcomes and process outcomes (use).

	1	2	3	4	5	
subitem not at all important	(•)	( )	( )	( )	( )	essential

Does your paper address subitem 22-i? *

Copy and paste relevant sections from the manuscript (include quotes in quotation marks "like this" to indicate direct quotes from your manuscript), or elaborate on this item by providing additional information not in the ms, or briefly explain why the item is not applicable/relevant for your study

no: not applicable, this is a protocol

22-ii) Highlight unanswered new questions, suggest future research

Highlight unanswered new questions, suggest future research.

	1	2	3	4	5	
subitem not at all important	(•)	( )	( )	( )	( )	essential

Does your paper address subitem 22-ii?

Copy and paste relevant sections from the manuscript (include quotes in quotation marks "like this" to indicate direct quotes from your manuscript), or elaborate on this item by providing additional information not in the ms, or briefly explain why the item is not applicable/relevant for your study

no: not applicable, this is a protocol

20) Trial limitations, addressing sources of potential bias, imprecision, and, if relevant, multiplicity of analyses

20-i) Typical limitations in ehealth trials

Typical limitations in ehealth trials: Participants in ehealth trials are rarely blinded. Ehealth trials often look at a multiplicity of outcomes, increasing risk for a Type I error. Discuss biases due to non-use of the intervention/usability issues, biases through informed consent procedures, unexpected events.

	1	2	3	4	5	
subitem not at all important	○	◉	○	○	○	essential

Does your paper address subitem 20-i? *

Copy and paste relevant sections from the manuscript (include quotes in quotation marks "like this" to indicate direct quotes from your manuscript), or elaborate on this item by providing additional information not in the ms, or briefly explain why the item is not applicable/relevant for your study

yes: The primary study limitations are loss to follow-up and missing
data points.

21) Generalisability (external validity, applicability) of the trial findings

NPT: External validity of the trial findings according to the intervention, comparators, patients, and care providers or centers involved in the trial

21-i) Generalizability to other populations

Generalizability to other populations: In particular, discuss generalizability to a general Internet population, outside of a RCT setting, and general patient population, including applicability of the study results for other organizations

	1	2	3	4	5	
subitem not at all important	○	◉	○	○	○	essential

Does your paper address subitem 21-i?

Copy and paste relevant sections from the manuscript (include quotes in quotation marks "like this" to indicate direct

quotes from your manuscript), or elaborate on this item by providing additional information not in the ms, or briefly explain why the item is not applicable/relevant for your study

Our research will provide gender- and age-stratified analyses,
as well as an understanding of the potential added benefits of
SMS support strategies alongside virtual reality and group problem management plus to inform differentiated HIV testing strategies among urban refugee and displaced youth, which can be adapted for diverse contexts.

21-ii) Discuss if there were elements in the RCT that would be different in a routine application setting

Discuss if there were elements in the RCT that would be different in a routine application setting (e.g., prompts/reminders, more human involvement, training sessions or other co-interventions) and what impact the omission of these elements could have on use, adoption, or outcomes if the intervention is applied outside of a RCT setting.

	1	2	3	4	5	
subitem not at all important	(•)	( )	( )	( )	( )	essential

Does your paper address subitem 21-ii?

Copy and paste relevant sections from the manuscript (include quotes in quotation marks "like this" to indicate direct quotes from your manuscript), or elaborate on this item by providing additional information not in the ms, or briefly explain why the item is not applicable/relevant for your study

"Retention is likely to be even lower outside of an RCT setting which included reminder sms and visits from peer navigators."

OTHER INFORMATION

23) Registration number and name of trial registry

Does your paper address CONSORT subitem 23? *

Copy and paste relevant sections from the manuscript (include quotes in quotation marks "like this" to indicate direct quotes from your manuscript), or elaborate on this item by providing additional information not in the ms, or briefly explain why the item is not applicable/relevant for your study

ClinicalTrials.gov NCT05187689
https://www.clinicaltrials.gov/ct2/show/NCT05187689?term=logie&draw=2&rank=2

24) Where the full trial protocol can be accessed, if available

Does your paper address CONSORT subitem 24? *

Cite a Multimedia Appendix, other reference, or copy and paste relevant sections from the manuscript (include quotes in quotation marks "like this" to indicate direct quotes from your manuscript), or elaborate on this item by providing additional information not in the ms, or briefly explain why the item is not applicable/relevant for your study

no because this is the full trial protocol

25) Sources of funding and other support (such as supply of drugs), role of funders

Does your paper address CONSORT subitem 25? *

Copy and paste relevant sections from the manuscript (include quotes in quotation marks "like this" to indicate direct quotes from your manuscript), or elaborate on this item by providing additional information not in the ms, or briefly explain why the item is not applicable/relevant for your study

This study is funded by the Canadian Institutes of Health Research (CIHR) and Grand Challenges Canada. Funding agencies played no role in the design or execution of the study. C. Logie is also funded by the Canada Research Chairs program (Tier 2: Logie), Canada Foundation for Innovation (Logie Lab), and the Ontario Ministry of Research and Innovation (ERA: Logie).

X27) Conflicts of Interest (not a CONSORT item)

X27-i) State the relation of the study team towards the system being evaluated

In addition to the usual declaration of interests (financial or otherwise), also state the relation of the study team towards the system being evaluated, i.e., state if the authors/evaluators are distinct from or identical with the developers/sponsors of the intervention.

	1	2	3	4	5	
subitem not at all important	○	⦿	○	○	○	essential

Does your paper address subitem X27-i?

Copy and paste relevant sections from the manuscript (include quotes in quotation marks "like this" to indicate direct quotes from your manuscript), or elaborate on this item by providing additional information not in the ms, or briefly explain why the item is not applicable/relevant for your study

yes

About the CONSORT EHEALTH checklist

As a result of using this checklist, did you make changes in your manuscript? *

- ○ yes, major changes
- ○ yes, minor changes
- ◉ no

What were the most important changes you made as a result of using this checklist?

How much time did you spend on going through the checklist INCLUDING making changes in your manuscript *

2 hours

As a result of using this checklist, do you think your manuscript has improved? *

- ○ yes
- ◉ no
- ○ Other:

Would you like to become involved in the CONSORT EHEALTH group?

This would involve for example becoming involved in participating in a workshop and writing an "Explanation and Elaboration" document

- ○ yes
- ◉ no
- ○ Other:

Any other comments or questions on CONSORT EHEALTH

This is not relevant for a protocol paper, half of these questions were irrelevant. It would be better to have a word document such as the consort is in, as easier to fill in.

STOP - Save this form as PDF before you click submit

To generate a record that you filled in this form, we recommend to generate a PDF of this page (on a Mac, simply select "print" and then select "print as PDF") before you submit it.

When you submit your (revised) paper to JMIR, please upload the PDF as supplementary file.

Don't worry if some text in the textboxes is cut off, as we still have the complete information in our database. Thank you!

Final step: Click submit !

Click submit so we have your answers in our database!

Submit

Never submit passwords through Google Forms.

Powered by Google Forms

This content is neither created nor endorsed by Google.

Report Abuse - Terms of Service - Additional Terms
